# Analysis of Bending Degree of Basilar Artery Using Image Processing

**DOI:** 10.3390/diagnostics12092066

**Published:** 2022-08-26

**Authors:** Jeehong Kim, Yeongmin Jang, Hyosung Kwak, Hilal Tayara, Kil To Chong

**Affiliations:** 1 Department of Renewable Energy Engineering, Jeonju Vision College, Jeonju 55069, Korea; 2 Department of Electronics and Information Engineering, Jeonbuk National University, Jeonju 54896, Korea; 3 Department of Diagnostic Radiology, Jeonbuk National University Medical School, Jeonju 54896, Korea; 4 School of International Engineering and Science, Jeonbuk National University, Jeonju 54896, Korea; 5 Advances Electronics and Information Research Center, Jeonbuk National University, Jeonju 54896, Korea

**Keywords:** basilar artery, bending, image processing, vector representations, angular calculation

## Abstract

The basilar artery, which is the core of the posterior circulation, supplies blood to the brainstem and cerebellum. When basilar artery blood circulation is impaired, several symptoms can occur. In addition, the bending of the basilar artery causes stroke and infarction. Therefore, an image processing method for analyzing the bending degree of the basilar artery is introduced herein. To analyze the bending degree, the coordinates of the center points of the basilar artery are extracted using image processing techniques such as Canny edge detection, the contour technique, and the distance conversion technique. An image reconstructed using the three-dimensional scatter plot function in MATLAB and vector plots is used to calculate the vectors for each central point of the basilar artery. Meanwhile, the angle of each central point is calculated by selecting the first central point where the basilar artery begins, the central point with the greatest bending degree, and the central point at which the branching ends. The greater the bending degree of the basilar artery is, the larger the magnitude of the vector in the bending direction. The obtained results are verified by experts in the field, and the proposed algorithm demonstrates good performance.

## 1. Introduction

The blood circulation system of the brain comprises an anterior circulation system and a basilar artery. The anterior circulation system comprises the anterior cerebral artery and the middle cerebral artery through the internal carotid artery and the posterior circulation system, which, in turn, comprises the posterior cerebral artery. The basilar artery connects the two vertebral arteries to one artery [1]. The basilar artery, which is the core of the posterior circulation, is formed when two vertebral arteries merge under the pons [2]. The basilar artery continues along the anterior surface of the brainstem and supplies blood to the brainstem and cerebellum [3]. When blood is not supplied accordingly to the brainstem and cerebellum, symptoms such as dizziness, dysarthria, diplopia, and tinnitus can occur [1,4]. These symptoms are known as vertebral basilar artery insufficiency [1,4]. Arteriosclerosis [5], thrombosis, arterial dissection, and vasculitis [6] are the main causes of vertebral basilar artery insufficiency. In addition to these symptoms, torsion of the basilar artery due to vertebral artery dominance results in ischemic stroke [7,8]. In addition, it was discovered that patients who exhibit changes in the morphology and geometry of the vertebral base system were more likely to develop stroke due to aneurysms and atherosclerosis [9]. Some studies have shown that blood flow decelerated by twisted blood vessels increases the possibility of microthrombus formation on the blood vessel wall [5,7]. Similarly, the bending of the basilar artery can compress and elongate the perforating artery of the pons, resulting in infarction [5,10]. In addition, as the bending of the basilar artery increases, the probability of compressive and/or stretching forces affecting the pons by perforating the branches increases, thereby increasing the risk of pons infarction [5,11]. With regard to other diseases associated with the basilar artery, when the basilar artery is dilated, twisted, or lengthened, which results in malformations, vertebrobasilar dolichoectasia may occur; in addition, spondylopathy can cause symptoms such as eye movement paralysis, trigeminal neuralgia, facial convulsions, paralysis, cerebral infarction, transient ischemic attack, hydrocephalus, brain stem compression, cerebral hemorrhage, and abrupt hearing loss by directly compressing the cranial nerves and brain stem [12,13,14,15,16]. These basilar arteries are located between several blood vessels that are near the cranial nerves and are associated closely with the adjacent regions of the cranial nerves, so they may be injured during neurosurgery [17]. Therefore, the bending degree of the basilar artery must be determined when performing neurosurgical operations. In this regard, the morphological changes in the basilar artery branch were analyzed in a study [18], whereas the geometric analysis of the basilar artery and the resulting blood flow were simulated in another study [17]. In addition, although we searched several other previous studies, it was confirmed that there was no study that numerically analyzed the basilar artery. The basilar artery is an important blood vessel for blood circulation in the brain. However, most diagnoses of the basilar artery are currently conducted based on the perspective and judgment of an expert via medical imaging. Misdiagnosis by experienced experts may occur because diseases with the same symptoms abound.

In this study, the bending degree of the basilar artery is analyzed via image processing, to reduce the probability of misdiagnosis and facilitate experts in performing diagnosis. The Department of Diagnostic Radiology, Jeonbuk National University Medical School, provided clinical data pertaining to the actual basilar artery, and the basilar artery was reconstructed using image processing techniques in MATLAB. For image processing, the basilar artery region was set as the region of interest for image processing, followed by Gaussian blurring to enhance the extraction, and the bone and brain cortex were removed using binarization to extract only the blood vessels from the noise-free image. Blood vessel data were obtained using the Canny edge detection technique, and the shape of the basilar artery was detected by connecting the blood vessel data extracted using the contour line technique. Furthermore, the data of the central point of the basilar artery were extracted via distance conversion. The extracted data were used to reconstruct the basilar artery in three dimensions using MATLAB’s three-dimensional (3D) scatter plot function. A study was conducted to analyze the bending degree of the basilar artery using angle measurements and vector plots on the reconstructed basilar artery plot. By measuring the angle of the basilar artery, the bending degree of the basilar artery was expressed as an angle, and the bending direction of the basilar artery was expressed as a vector plot, such that it can be interpreted intuitively. In addition, the bending direction was expressed graphically to facilitate the analysis. The proposed method provides an automated and accurate method for analyzing the bending of the basilar artery, which is an alternative to using an MRI image only, to enable specialists in performing diagnosis [5,19,20,21]. In addition, this method enables the accurate prediction of the potential risk and can be used extensively in basic research, as well as in future treatments and preventions. The obtained results were verified by field experts. The remainder of this paper is organized as follows. Section 2 introduces the data acquisition and processing of the basilar artery. The method to calculate the bending degree of the basilar artery is described in Section 3. The results of this study are presented in Section 4. Finally, the proposed method and the results obtained are discussed in Section 5.

## 2. Basilar Artery Data

**Figure 1:**
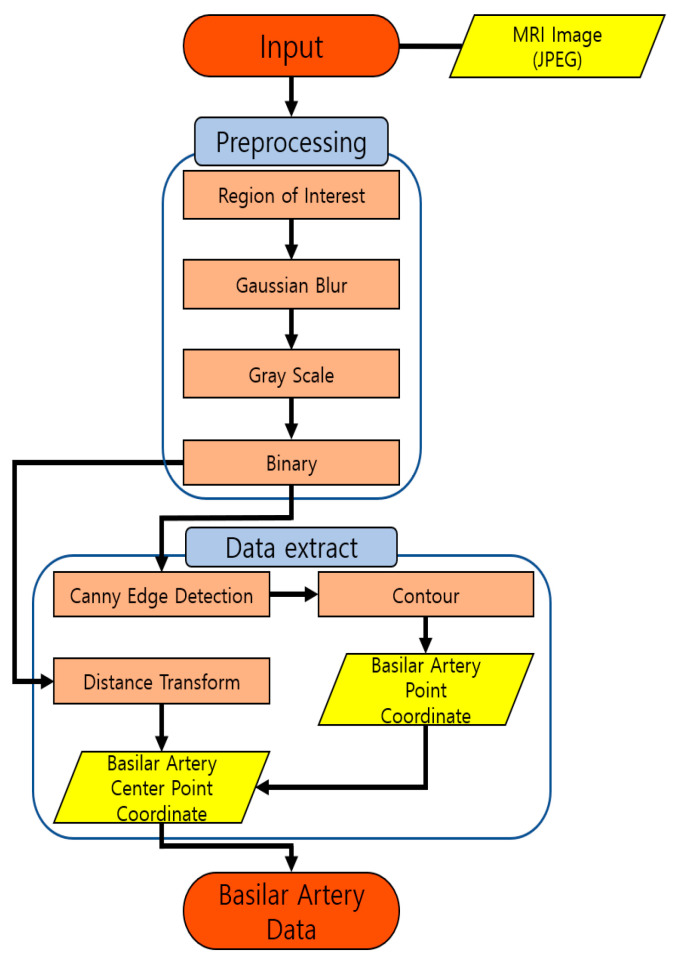
Basilar artery data-extraction flowchart.

In this study, clinical data pertaining to the basilar artery from the Department of Diagnostic Radiology, Medical School, Jeonbuk National University, are used. These data were extracted by converting an MRI image in the Digital Imaging and Communications in Medicine (DICOM) format into a Joint Photographic Experts Group (JPEG) image. Subsequently, Figure 1 shows the algorithm for extracting basilar artery data from the extracted JPEG-format MRI image. In the preprocessing step, the region of interest of the basilar artery was selected, and Gaussian blurring was performed to remove the noise that may occur during image conversion. The blood vessels were extracted only via grayscale conversion and binary conversion. In the data-extraction step, the basilar artery was detected using the Canny edge detection algorithm, and the shape of the basilar artery was detected by connecting the detected edges using the contour method. Furthermore, the central points of the basilar artery were detected using the distance conversion technique. The details regarding these two steps are described in the following sections.

### 2.1. Preprocessing

The region of interest of the basilar artery was set from the point where the vertebral arteries on both sides coincided to the point where the basilar artery branched off. The size of a person’s head and the location of the basilar artery were different for each dataset, therefore, they were set manually. The noise in the extracted region of interest was removed using a Gaussian filter, as mathematically represented by Equation (1) [22]

(1) $$G\left(x,y\right)=\frac{1}{2\pi \sigma^{2}}e^{-\left(\frac{x^{2}+y^{2}}{2\sigma^{2}}\right)},$$

where (*x*,*y*) is the pixel position of the image, and σ is the standard deviation that determines the width of the Gaussian distribution mask and the smoothing degree. A greater σ implies a better smoothing effect. In this study, this value was set to 1.5 empirically, and the size of the two-dimensional Gaussian filter mask was set automatically based on the σ value specified. The region of interest from which noise was removed using the Gaussian filter was subjected to grayscale and binary transformation to remove unnecessary brain cortex and bones. When a high threshold value was set, the blood vessels extracted were smaller than the actual vessels, and when a low threshold value was set, unnecessary data such as those from the bones and brain cortex were extracted simultaneously. Therefore, to remove unnecessary data and to extract the blood vessels accurately, the threshold value for binary conversion was set empirically to 180 [23].

### 2.2. Data Extraction

#### 2.2.1. Basilar Artery Edge Extraction

The edges of the basilar artery were detected using the Canny edge detection algorithm. An edge is a boundary between objects constituting an image and includes information such as the position, size, and direction of an object in the digital image [24,25]. Canny’s criteria for a good edge should exhibit a low error rate and an accurate location [26], i.e., the distance between the detected edge and the edge of the image should be the minimum. Finally, for a single-edge response, only one edge from the edges in the image need to be detected. The Canny edge detection algorithm was processed in five steps [27]. To remove noise, blurring was performed. The horizontal and vertical edges were detected using the Sobel operator, and the magnitude and direction of the gradient were calculated [22]. Using non-maximum suppression, the pixel with the local maximum was set as the edge [22]. The set edge was determined as the final edge using two thresholds: the edge above the high threshold was determined, and the edge with a value between the low and high threshold values was considered a valid edge, by using the hysteresis edge-tracking technique.

To connect the edges of the detected basilar artery, regions having the same pixel value were connected using the contour method. The contour line technique connects continuous data with the same value and can be used for shape analysis, object detection, and recognition [28]. We used the contour method to extract data and coordinates for the basilar artery morphology. The hierarchy is important for obtaining the contours. Objects in the image can appear in different locations, in which case some shapes may be inside other shapes. In this case, the data outside are known as the parent hierarchy, and the data inside are known as the child hierarchy. In this study, because the edges of the blood vessels were pixel data, we obtained all the contour lines by constructing all the hierarchies, used an approximation method that returns only points that allowed us to construct the contour lines to save memory, and finally constructed the contours of the basilar artery.

#### 2.2.2. Basilar Artery Center Point Extraction

The center points of the basilar artery were detected using a distance transformation technique. Distance transformation is a method of expressing the distance from an object to the non-feature pixels as the shortest distance [29]. We determined the distance between the basilar artery and the background and then identified the center point of the largest pixel value [30].

## 3. Calculations of Bending Degree of Basilar Artery and Angle

### 3.1. Calculation of Bending Degree of Basilar Artery

**Figure 2:**
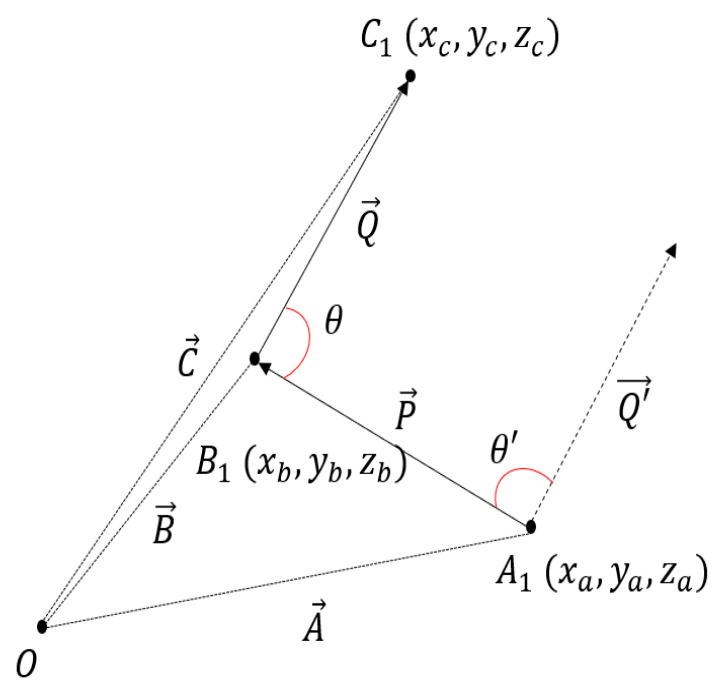
Definition of $-\vec{P}$ and $\vec{Q^{\prime }}$ for degree of bending analysis.

After calculating the vector for the center points of the extracted basilar artery data using the vector plot in MATLAB, the angle between the two vectors was defined as θ, and the value of $\theta^{\prime }$ was calculated to analyze the bending degree of the basilar artery. The dot product of the vector was used to calculate the value of $\theta^{\prime }$. Equation (2) and Figure 2 shows the expression of the dot product of vectors for determining $\theta^{\prime }$ [31].

(2) $$\vec{P}=\vec{B}-\vec{A},\;\;\vec{Q}=\vec{C}-\vec{B}\;.\;\theta^{\prime }=\frac{\vec{Q^{\prime }}\cdot \left(-\vec{P}\right)}{\left|\vec{Q^{\prime }}\right|\left|\left(-\vec{P}\right)\right|}=\frac{\left(\vec{C}-\vec{B}\right)\cdot \left(\vec{A}-\vec{B}\right)}{\left|\vec{C}-\vec{B}\right|\left|\vec{A}-\vec{B}\right|}.$$

The bending degree θ′ was calculated using the dot product of the angle vector between the two vectors. It is assumed that $A_{1}\left(x_{a},\;y_{a},\;z_{a}\right)$ is the first center point of the basilar artery on the z-axis, and the distance from the origin to $A_{1}$ is defined as $\vec{A}$. Furthermore, it is assumed that the second center point is $B_{1}\left(x_{b},\;y_{b},\;z_{b}\right)$, and the distance from the origin to $B_{1}$ is defined as $\vec{B}$. Additionally, it is assumed that the third central point is $C_{1}\left(x_{c},\;y_{c},\;z_{c}\right)$, and the distance from the origin to $C_{1}$ is defined as $\vec{C}$. The distance from $A_{1}$ to $B_{1}$ is defined as $\vec{P}$, and the value of $\vec{P}$ can be obtained by subtracting $\vec{A}$ from $\vec{B}$. The distance from $B_{1}$ to $C_{1}$ is defined as $\vec{Q}$, and the value of $\vec{Q}$ is calculated by subtracting $\vec{B}$ from $\vec{C}$. When the origin is shifted to $A_{1},$ and $\vec{Q}$ is moved to the origin, assuming $\vec{P}$ is $-\vec{P}$ and $\vec{Q}$ is $\vec{Q^{\prime }}$, the formula for the dot product of $-\vec{P}$ and $\vec{Q^{\prime }}$ is as shown in Equation (2).

### 3.2. Calculation of Angle of Basilar Artery

**Figure 3:**
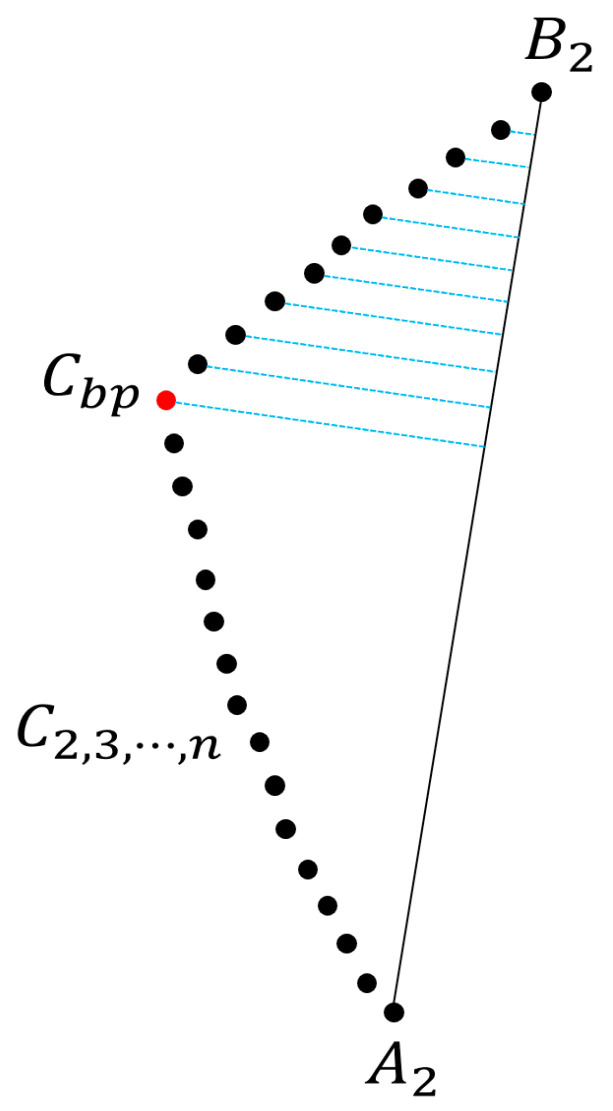
Center point of basilar artery with greatest bending degree calculated based on normal line.

As shown in Figure 3, we define the point where both vertebral arteries merge as the first point $A_{2}$ of the center points of the basilar artery, and the point where the basilar artery branches off as $B_{2}$; subsequently, we drew a line to connect $A_{2}$ and $B_{2}$. We define the center points between $A_{2}$ and $B_{2}$ as $C_{2,3,\ldots ,n}$, connect virtual normals to each point based on Equation (3), and define the point with the normal possessing the longest length as $C_{bp}$; subsequently, we drew two lines to connect $C_{bp}$ and $A_{2}$ and $C_{bp}$ and $B_{2}$, respectively

(3) $$a=A_{2}-B_{2},\;\;b=C_{2,3,\ldots ,n}-B_{2}\;\;d=\frac{\sqrt{\sum {\left(\vec{a}⊗\vec{b}\right)}^{2}}}{\sqrt{\sum {\vec{a}}^{2}}},$$

where *d* is the length of each normal line to the line connecting $A_{2}$ to $B_{2}$ at each point of $C_{2,3,\cdots ,n}$. In other words, it can be concluded that the center point with the highest value of d is the center point with the highest bending degree.

## 4. Results

The proposed algorithm was evaluated based on the data of 53 patients from the Jeonbuk National University Hospital. Herein, we provide the analysis of basilar artery bending based on a total of four cases: two cases in which the bending of the basilar artery is normal, one case in which the bending is in progress, and one case in which the bending is severe. The analysis results of all cases were verified by K.H.S., an expert in the Department of Radiology, Jeonbuk National University Hospital.

**Figure 4:**
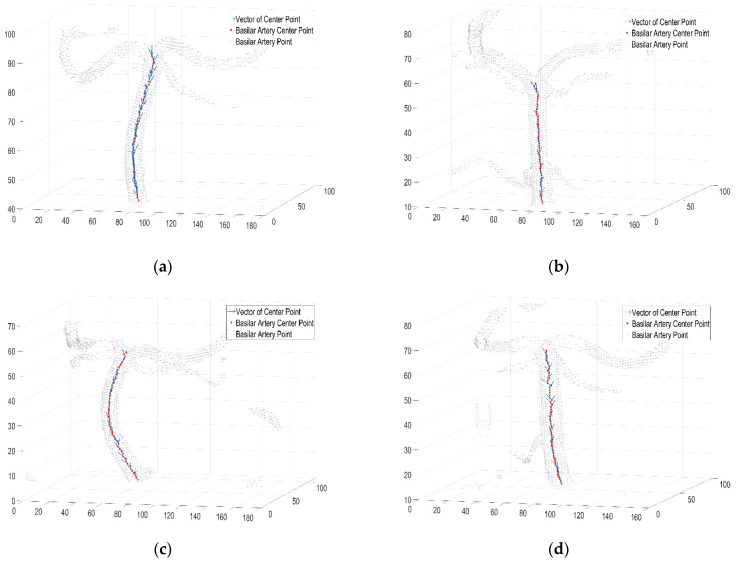
(**a**–**d**) Vector plot of bending degree of basilar artery calculated as dot product of vectors. (**a**) person A; (**b**) person B; (**c**) person C; (**d**) person D (size).

In the vector plot of Figure 4, the bending degree of the basilar artery is represented as a vector of the central points of the basilar artery. The greater the magnitude of the vector is, the greater the bending degree. In addition, in the vector direction, it was confirmed that the basilar artery was bent along the coronal plane axis, and the basilar artery was extended along the sagittal plane axis. As shown in Figure 4a, bending and elongation occurred in the direction of the vector in the coronal and sagittal axes, and, considering the magnitude of the vector, it was observed that the bending degree progressed to some extent. As shown in Figure 4b,d, the direction and magnitude of the vector are shown by the sagittal axis, which implies that the basilar artery was elongated along the sagittal axis, indicating that the bending degree of the basilar artery was relatively normal. 

**Figure 5:**
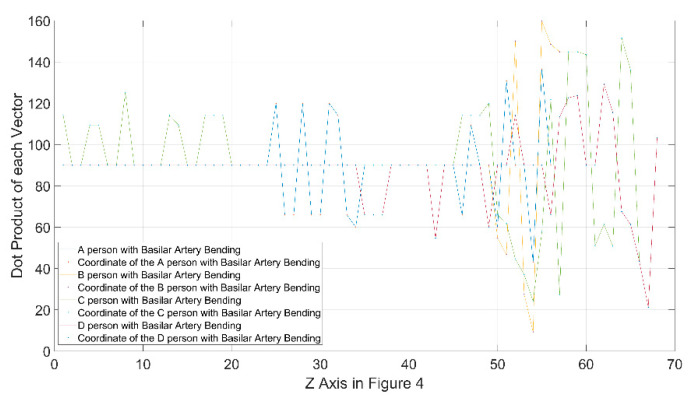
Graph showing bending degree of basilar artery calculated as dot product of vector (size).

As shown, the direction and magnitude of the vector in Figure 4c were elongated along the sagittal plane axis, and the bending degree in the coronal plane axis was high. It was observed that the direction of the vector continued to appear based on the sagittal and coronal planes. This implies that the bending degree of the basilar artery (Figure 4c) was high. The dot products of the vectors of the center points of the basilar artery in Figure 4 are expressed graphically in Figure 5. In Figure 5, the value of the x-axis is the value of the z-axis in Figure 4, and the value of the y-axis is the dot product of the vectors. It can be confirmed that the graphs of B and D were normal, and that a portion with a certain bending degree appeared in A. Additionally, it can be confirmed that bending occurred in a portion branching into the posterior cerebral artery. Finally, it was observed that C exhibited a severe degree of bending.

**Figure 6:**
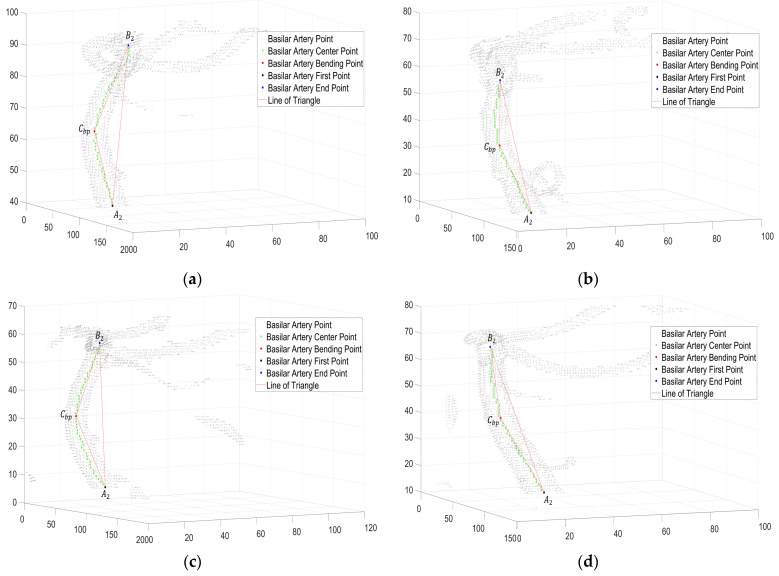
Angle measurement plot of basilar artery. (**a**) Person A; (**b**) Person (B); (**c**) Person C; (**d**) Person D (size).

Figure 6 shows the bending degree of the basilar artery, and the order is the same as that shown in Figure 4. As described earlier, $A_{2}$ is the first point in Figure 6, $B_{2}$ is the end point, and $C_{bp}$ is the bending point. Like Figure 4, it was observed that each basilar artery was bent along the coronal plane or the sagittal plane; furthermore, the higher the bending degree was, the smaller the angle of $C_{bp}$. In addition, each point of the basilar artery determined by the proposed algorithm was accurate, as confirmed by an expert. Table 1 summarizes the angles of the bending degree, as shown in Figure 6. The results of Person E to Person BA are shown in Supplementary file. A video explaining the results is shown in Supplementary file.

## 5. Conclusions

In this study, we analyzed the bending degree of the basilar artery via image processing and the vector dot product. Although we searched several previous studies related to the basilar artery, there was no study that numerically analyzed the basilar artery. Therefore, as most diagnoses rely only on the experience of experts, the probability of misdiagnosis is high. Therefore, in this study, image processing techniques were used. By analyzing the bending degree of basilar arteries using the proposed algorithm, basilar arteries with relatively normal bending, advanced bending, and severe bending can be identified numerically. However, when the vector dot product change in the cylindrical coordinate system was expressed as a two-dimensional graph, it was confirmed that the representation of the dot product direction was limited. Since the coordinates of the extracted center point were fixed coordinates, the measured angle did not change even when the basilar artery was rotated. In fact, it was fixed as the angle of the sagittal axis. As a future study, we plan to conduct a morphological analysis study that can more intuitively grasp the shape of blood vessels, through the analytical study of more accurate vector change and coordinate transformation. 

## Figures and Tables

**Table 1:** Measurement of angle of basilar artery.

Person	Bending Angle	Person	Bending Angle	Person	Bending Angle
A	135.0	S	153.6	AK	86.1
B	146.0	T	118.7	AL	166.5
C	114.2	U	122.1	AM	120.5
D	156.5	V	162.6	AN	144.7
E	146.6	W	94.1	AO	142.0
F	122.8	X	161.5	AP	134.3
G	112.8	Y	102.0	AQ	148.8
H	78.6	Z	101.8	AR	107.9
I	107.9	AA	71.0	AS	109.3
J	107.8	AB	101.3	AT	127.3
K	133.5	AC	108.4	AU	135.6
L	136.8	AD	110.2	AV	127.5
M	67.1	AE	156.2	AW	92.0
N	136.0	AF	68.9	AX	111.6
O	111.8	AG	114.0	AY	107.4
P	133.8	AH	111.9	AZ	120.5
Q	117.9	AI	115.1	BA	118.0
R	119.2	AJ	157.5		

## Data Availability

Not applicable.

## Supplementary Materials

The following supporting information can be downloaded at: https://www.mdpi.com/article/10.3390/diagnostics12092066/s1. Figure S1: Angle measurement plot of basilar artery of Person E to person BA. Video S1: Illustration of the proposed algorithm.
