# Supplementary material for: Analysis of Bending Degree of Basilar Artery Using Image Processing
Source: Diagnostics (Basel). 2022 Aug 26;12(9):2066. doi: 10.3390/diagnostics12092066 (PMC9498019; doi:10.3390/diagnostics12092066)
Supplement: Supplementary file 1 [file diagnostics-12-02066-s001.zip › diagnostics-1814175-supplementary/diagnostics-1814175-supplementary.pdf]

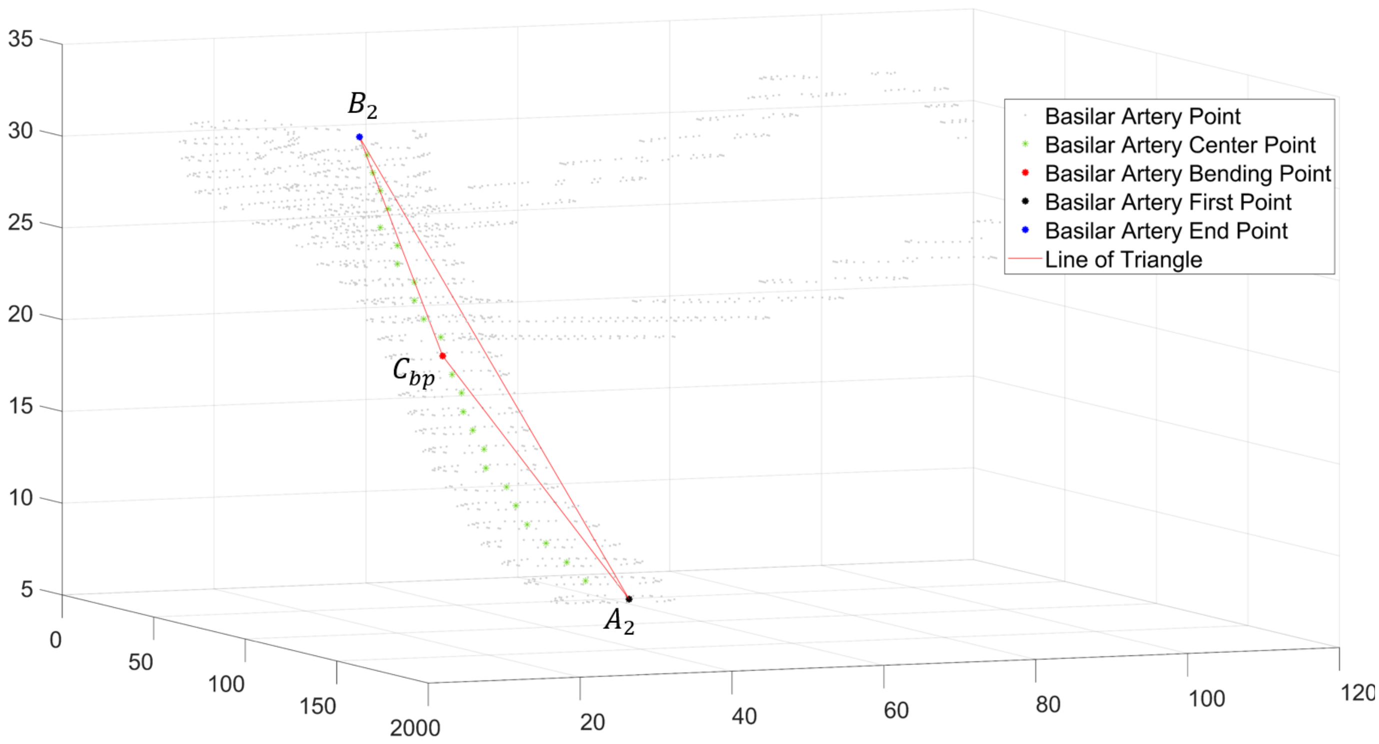
Basilar Artery Point
Basilar Artery Center Point
Basilar Artery Bending Point
Basilar Artery First Point
Basilar Artery End Point
Line of Triangle
B_2
C_{bp}
A_2
35
30
25
20
15
10
5
0
50
100
150
2000
20
40
60
80
100
120

(a)

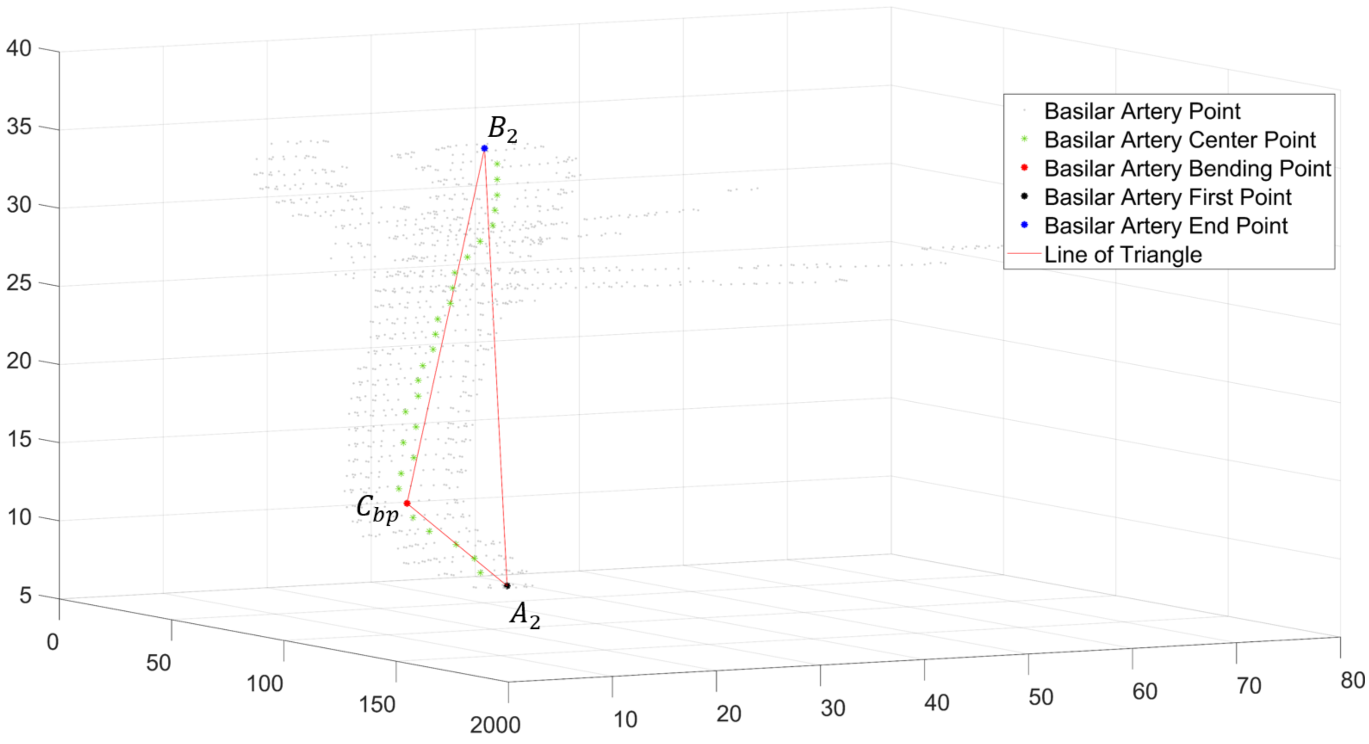
Basilar Artery Point
Basilar Artery Center Point
Basilar Artery Bending Point
Basilar Artery First Point
Basilar Artery End Point
Line of Triangle
B_2
C_{bp}
A_2
40
35
30
25
20
15
10
5
0
50
100
150
2000
10
20
30
40
50
60
70
80

(b)

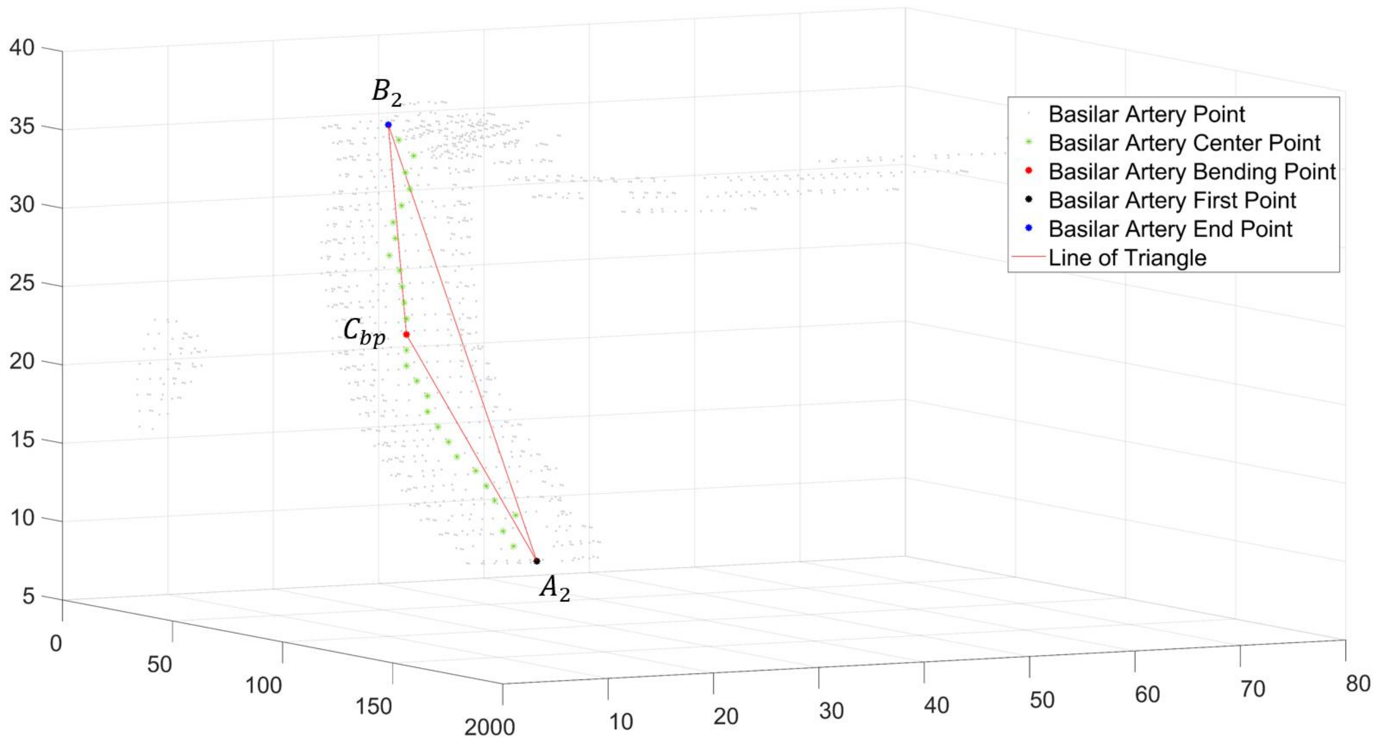

40
35
30
25
20
15
10
5
0
50
100
150
2000
10
20
30
40
50
60
70
80
B_2
C_{bp}
A_2
Basilar Artery Point
Basilar Artery Center Point
Basilar Artery Bending Point
Basilar Artery First Point
Basilar Artery End Point
Line of Triangle

(c)

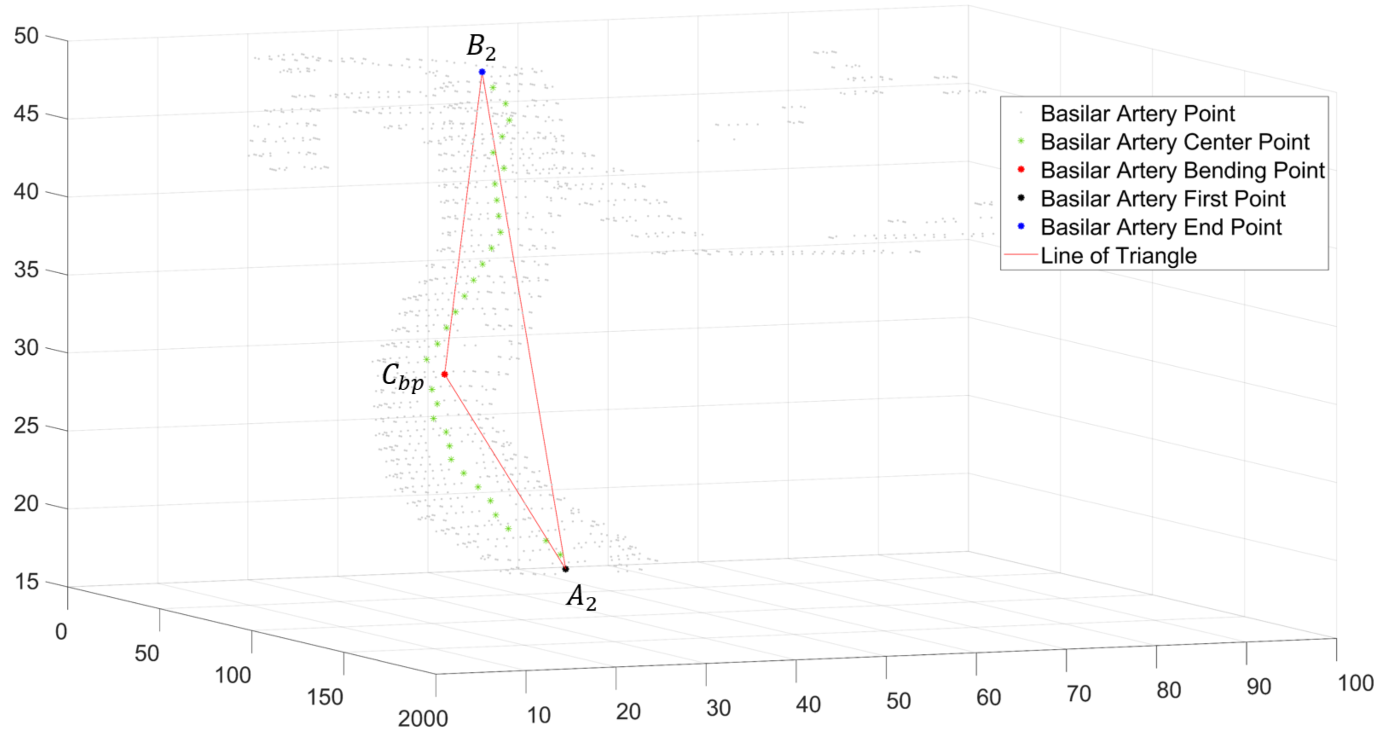

50
45
40
35
30
25
20
15
0
50
100
150
2000
10
20
30
40
50
60
70
80
90
100
B_2
C_{bp}
A_2
Basilar Artery Point
Basilar Artery Center Point
Basilar Artery Bending Point
Basilar Artery First Point
Basilar Artery End Point
Line of Triangle

(d)

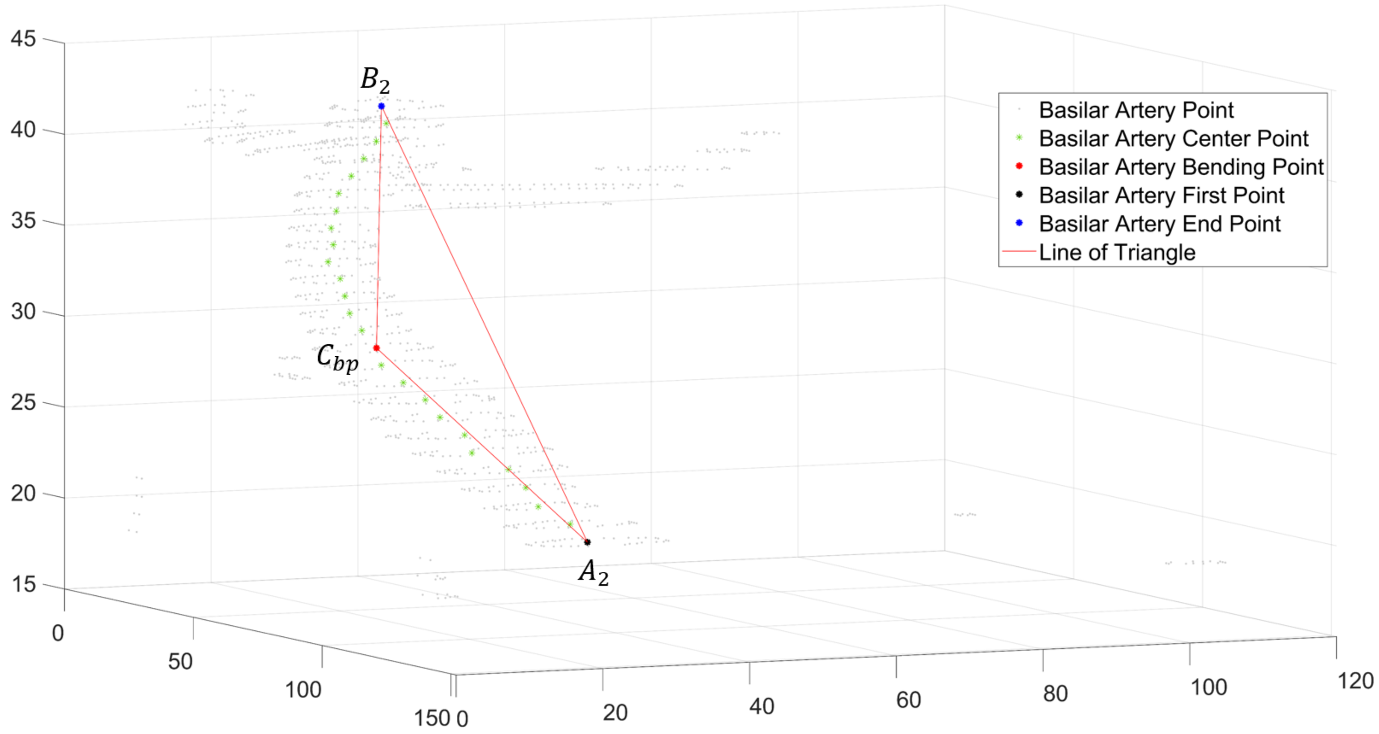

Basilar Artery Point
Basilar Artery Center Point
Basilar Artery Bending Point
Basilar Artery First Point
Basilar Artery End Point
Line of Triangle
B_2
C_{bp}
A_2

(e)

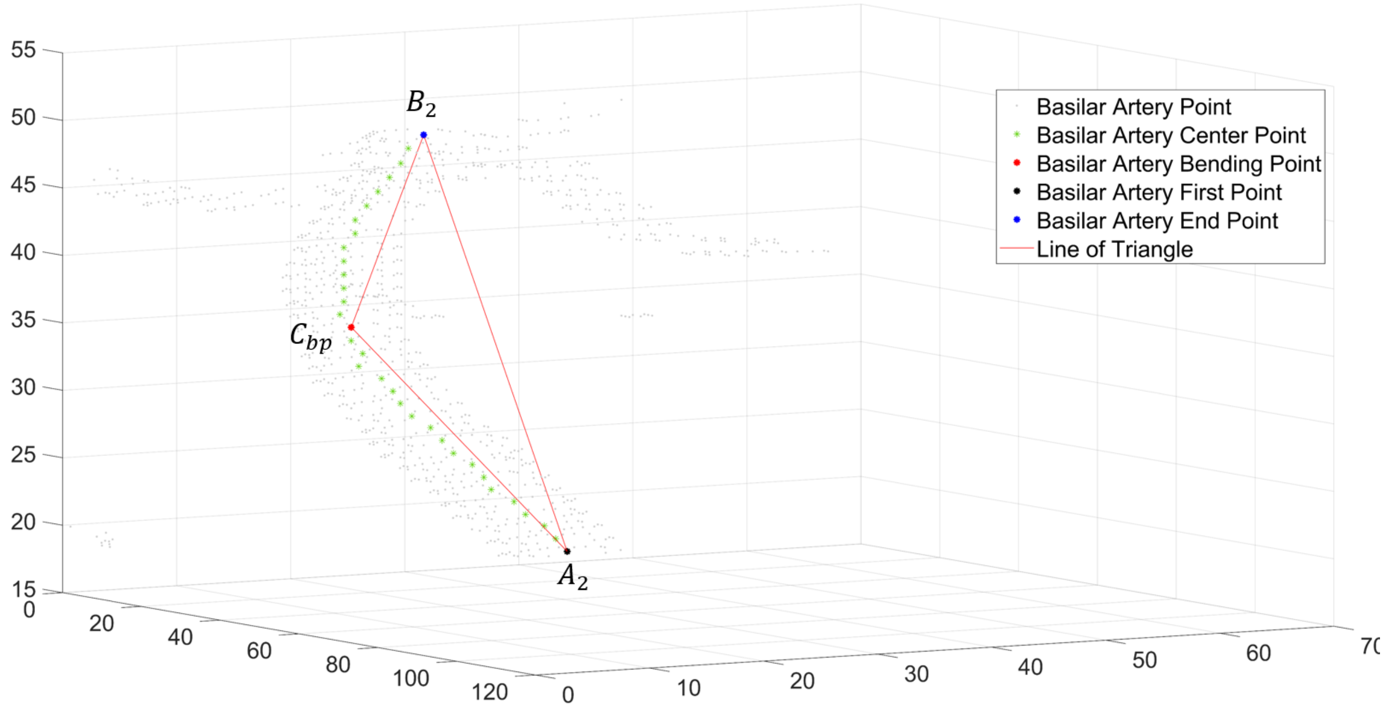

Basilar Artery Point
Basilar Artery Center Point
Basilar Artery Bending Point
Basilar Artery First Point
Basilar Artery End Point
Line of Triangle
B_2
C_{bp}
A_2

(f)

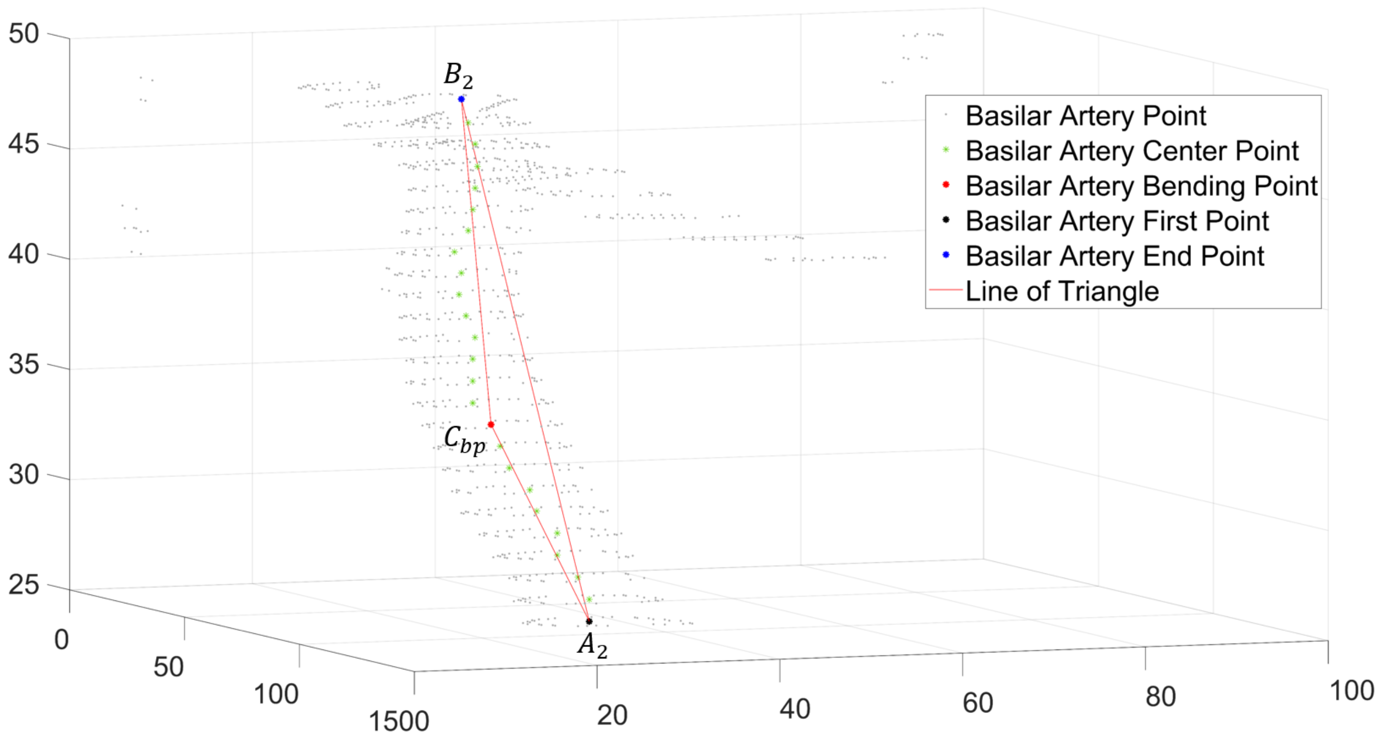

50
45
40
35
30
25
0
50
100
1500
20
40
60
80
100
B_2
C_{bp}
A_2
Basilar Artery Point
Basilar Artery Center Point
Basilar Artery Bending Point
Basilar Artery First Point
Basilar Artery End Point
Line of Triangle

(g)

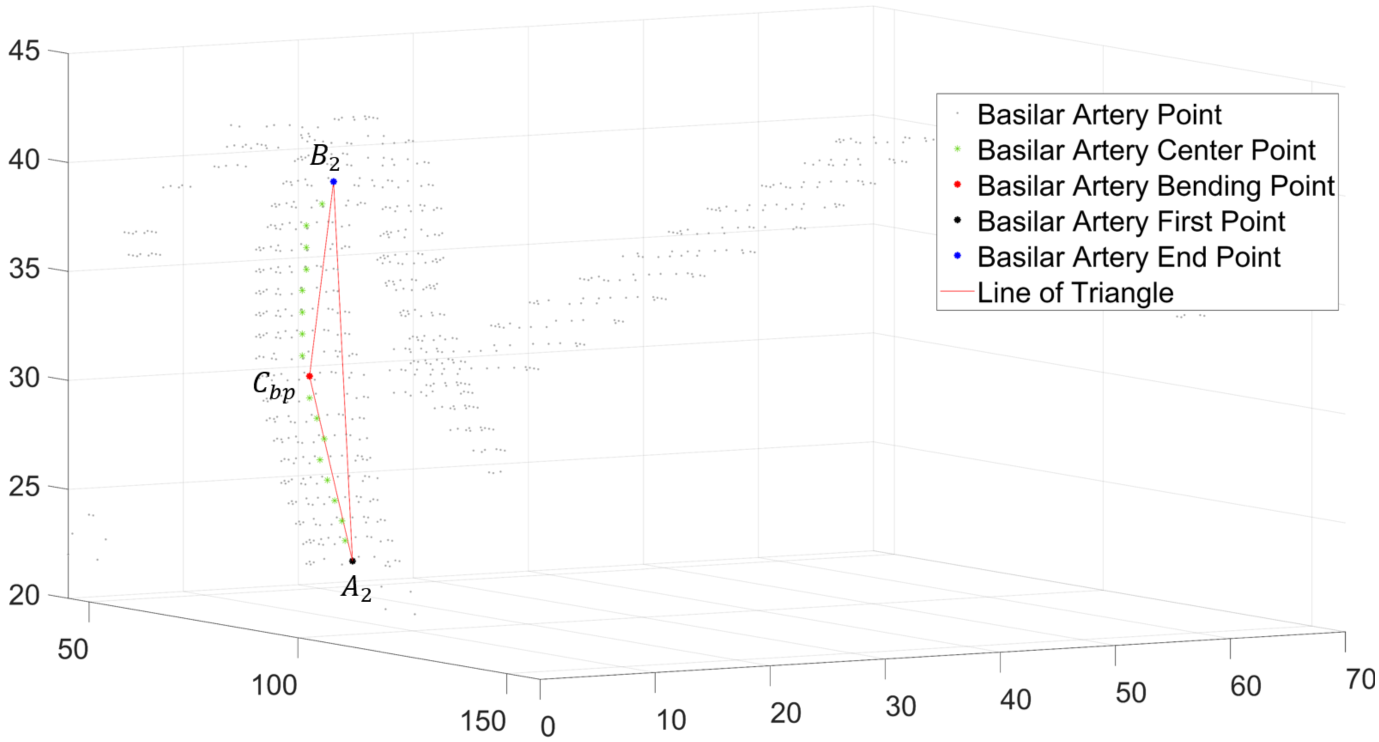

45
40
35
30
25
20
50
100
150
0
10
20
30
40
50
60
70
B_2
C_{bp}
A_2
Basilar Artery Point
Basilar Artery Center Point
Basilar Artery Bending Point
Basilar Artery First Point
Basilar Artery End Point
Line of Triangle

(h)

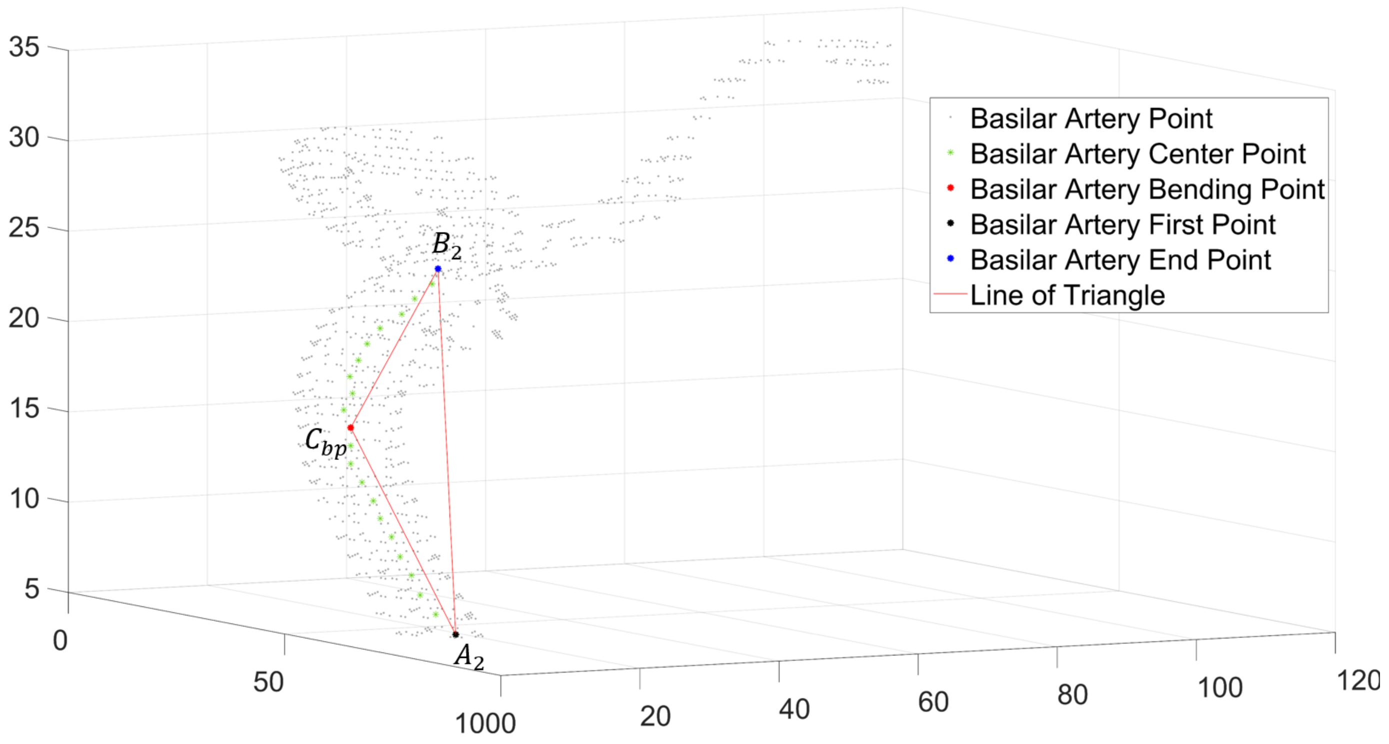
Basilar Artery Point
Basilar Artery Center Point
Basilar Artery Bending Point
Basilar Artery First Point
Basilar Artery End Point
Line of Triangle
B_2
C_{bp}
A_2
35
30
25
20
15
10
5
0
50
1000
20
40
60
80
100
120

(i)

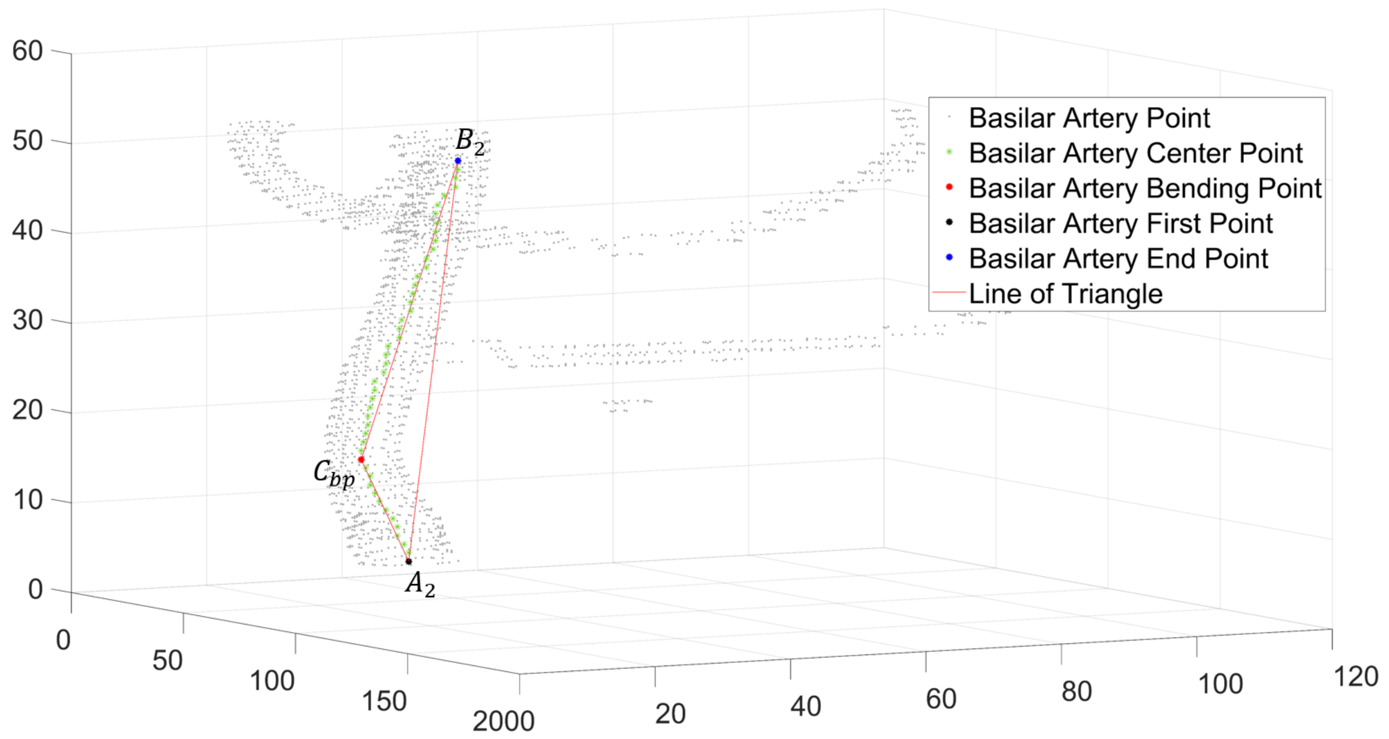
Basilar Artery Point
Basilar Artery Center Point
Basilar Artery Bending Point
Basilar Artery First Point
Basilar Artery End Point
Line of Triangle
B_2
C_{bp}
A_2
60
50
40
30
20
10
0
0
50
100
150
2000
20
40
60
80
100
120

(j)

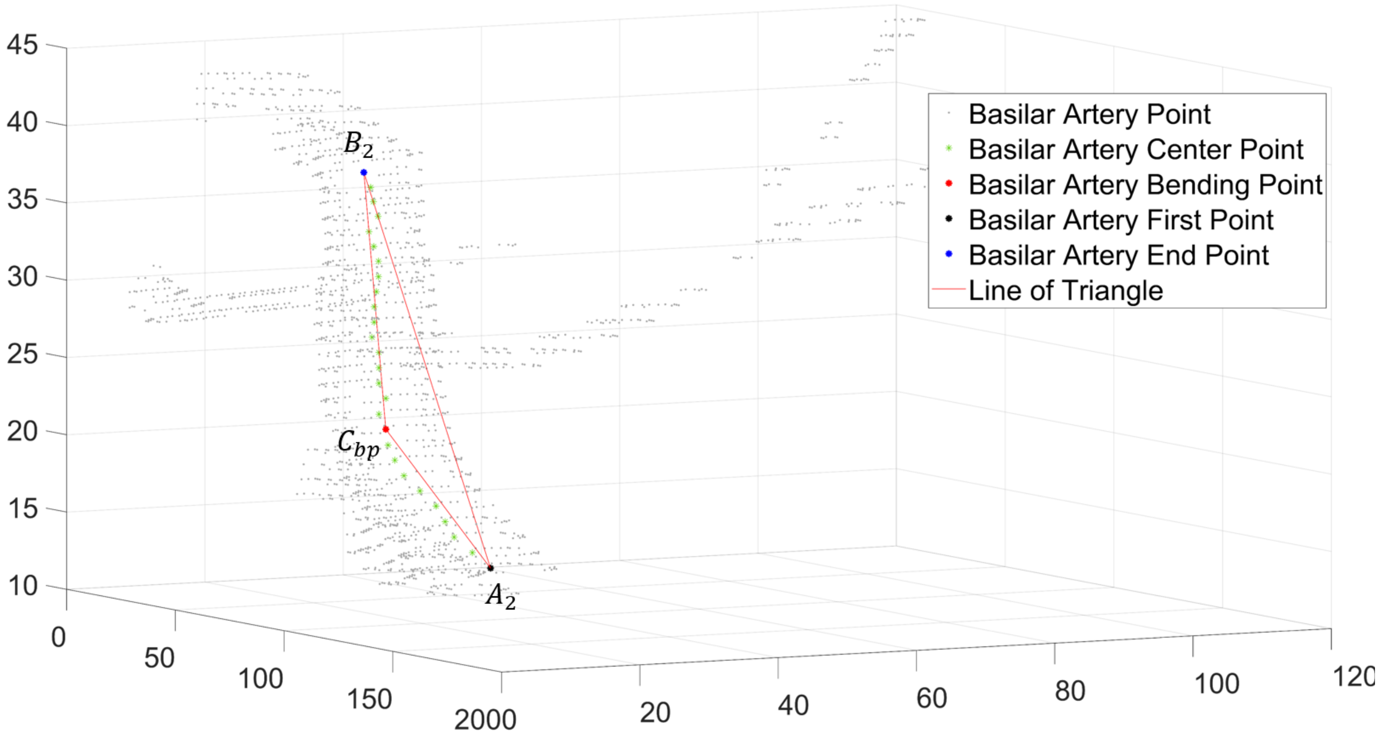
45
40
35
30
25
20
15
10
0
50
100
150
2000
20
40
60
80
100
120
B2
Cbp
A2
Basilar Artery Point
Basilar Artery Center Point
Basilar Artery Bending Point
Basilar Artery First Point
Basilar Artery End Point
Line of Triangle

(k)

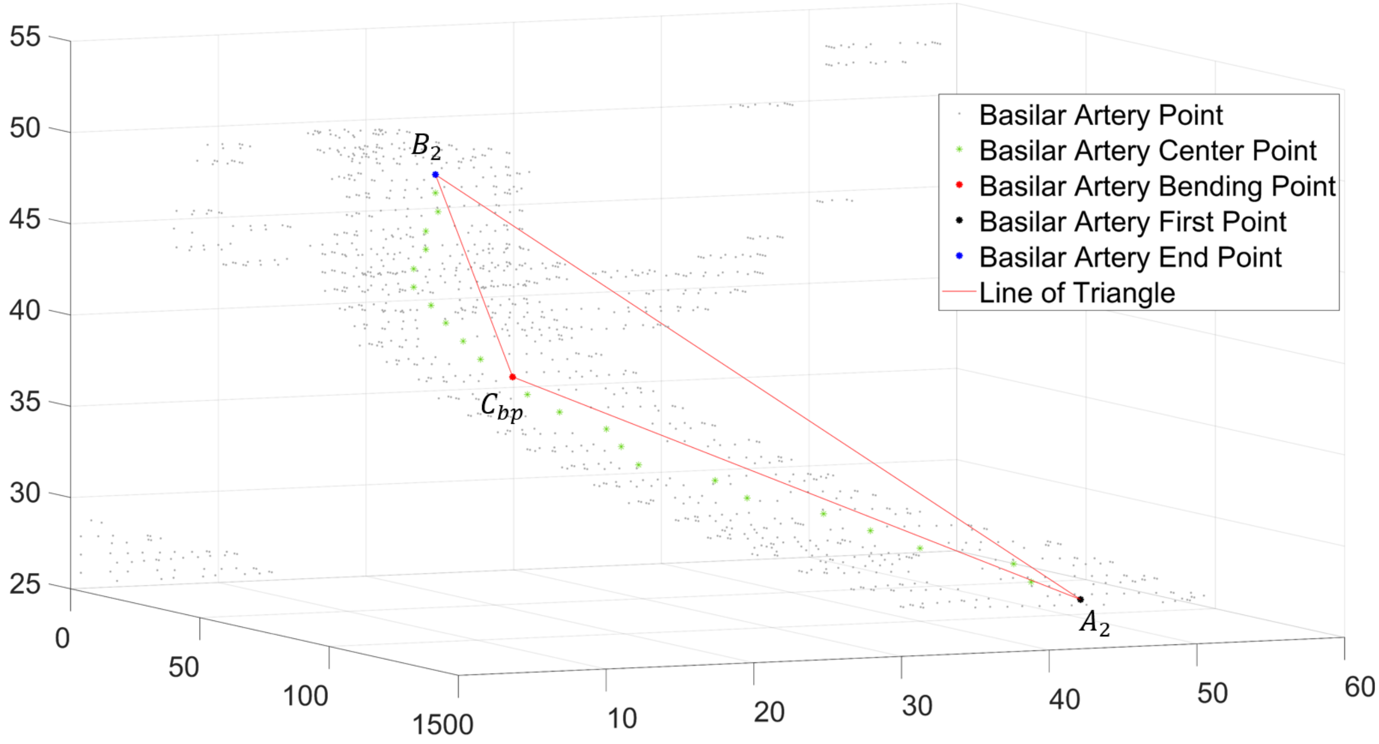
55
50
45
40
35
30
25
0
50
100
1500
10
20
30
40
50
60
B2
Cbp
A2
Basilar Artery Point
Basilar Artery Center Point
Basilar Artery Bending Point
Basilar Artery First Point
Basilar Artery End Point
Line of Triangle

(l)

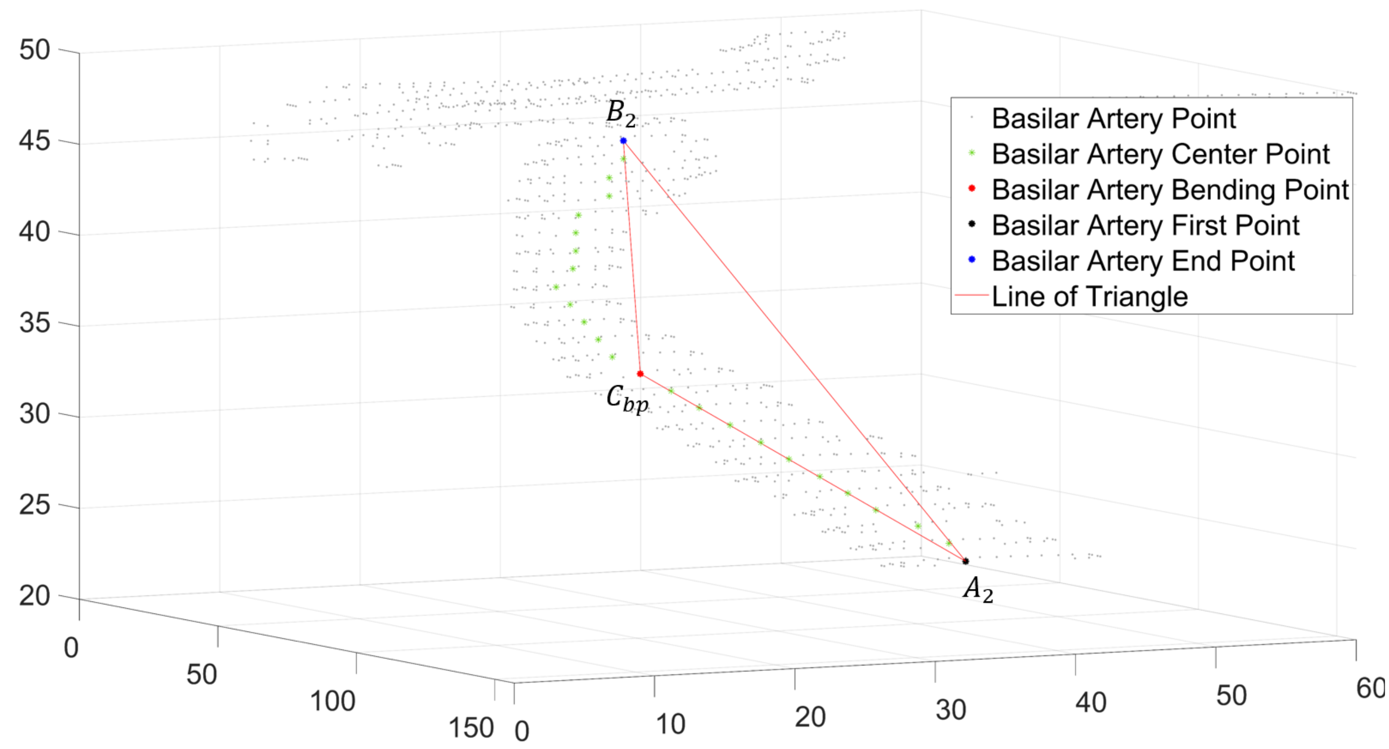
50
45
40
35
30
25
20
0
50
100
150
0
10
20
30
40
50
60
B_2
C_{bp}
A_2
Basilar Artery Point
Basilar Artery Center Point
Basilar Artery Bending Point
Basilar Artery First Point
Basilar Artery End Point
Line of Triangle

(m)

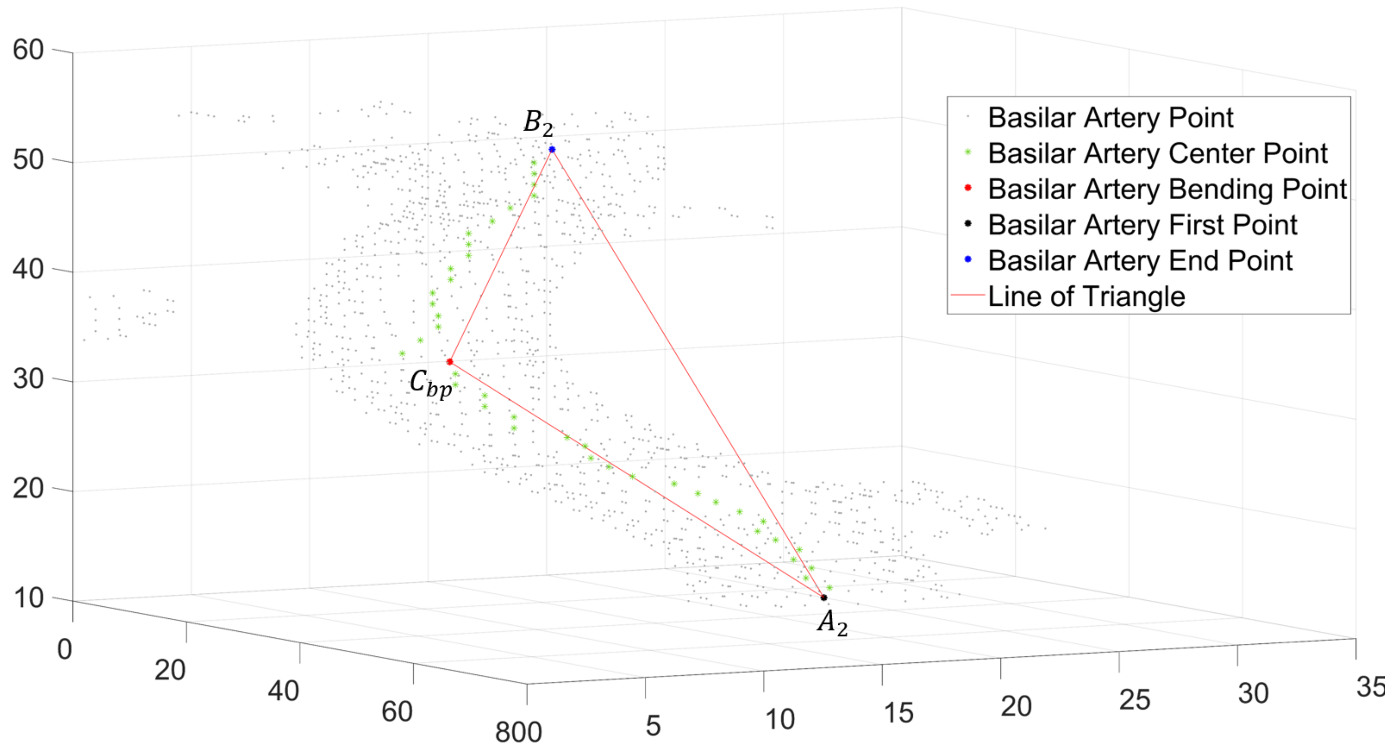
60
50
40
30
20
10
0
20
40
60
800
5
10
15
20
25
30
35
B_2
C_{bp}
A_2
Basilar Artery Point
Basilar Artery Center Point
Basilar Artery Bending Point
Basilar Artery First Point
Basilar Artery End Point
Line of Triangle

(n)

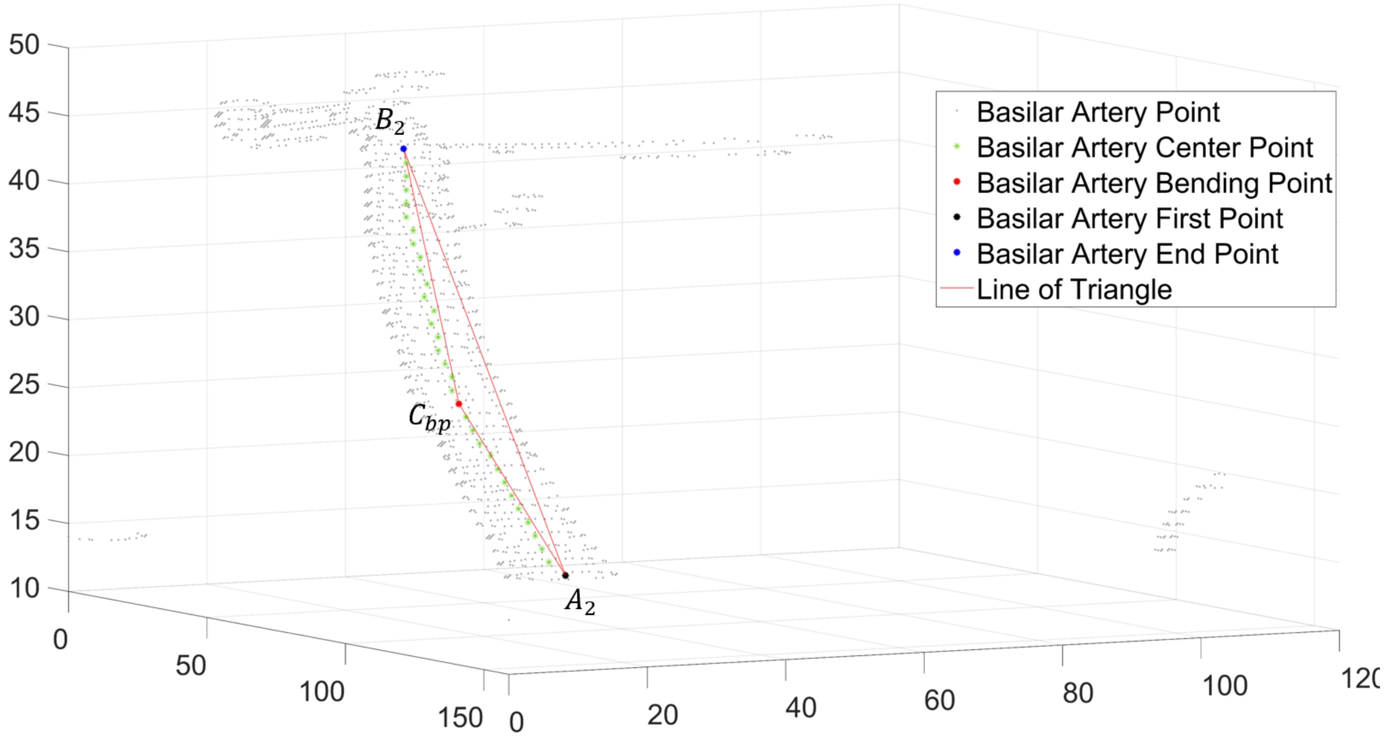

50
45
40
35
30
25
20
15
10
0
50
100
150
0
20
40
60
80
100
120
B_2
C_{bp}
A_2
Basilar Artery Point
Basilar Artery Center Point
Basilar Artery Bending Point
Basilar Artery First Point
Basilar Artery End Point
Line of Triangle

(o)

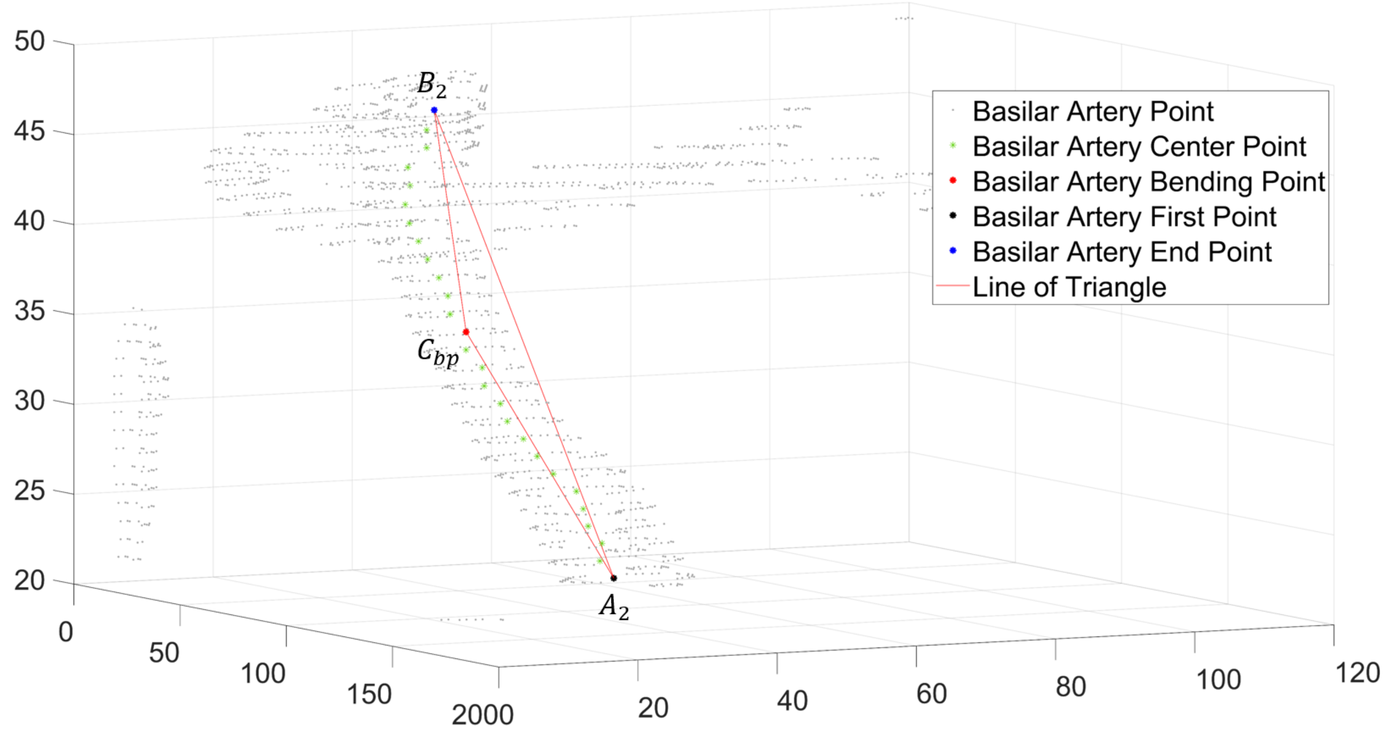

50
45
40
35
30
25
20
0
50
100
150
200
0
20
40
60
80
100
120
B_2
C_{bp}
A_2
Basilar Artery Point
Basilar Artery Center Point
Basilar Artery Bending Point
Basilar Artery First Point
Basilar Artery End Point
Line of Triangle

(p)

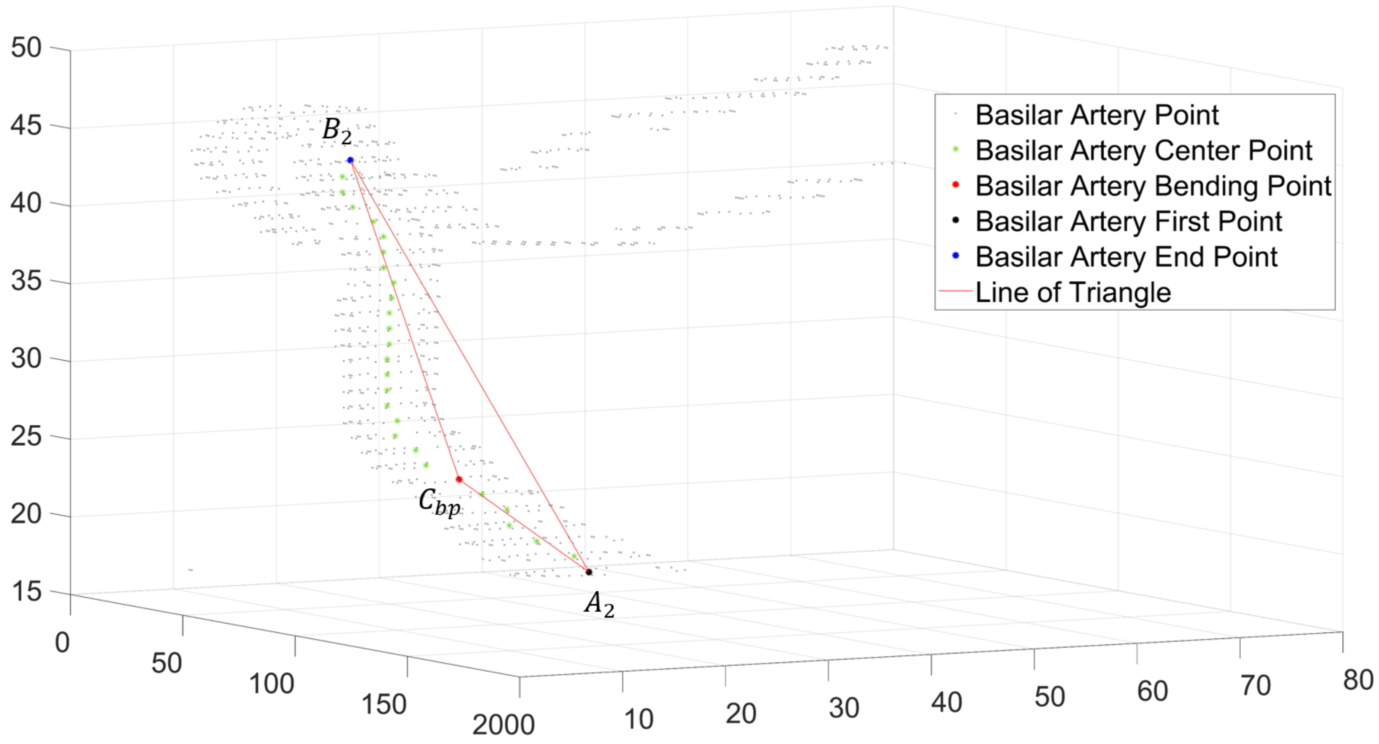
Basilar Artery Point
Basilar Artery Center Point
Basilar Artery Bending Point
Basilar Artery First Point
Basilar Artery End Point
Line of Triangle
B_2
C_{bp}
A_2

(q)

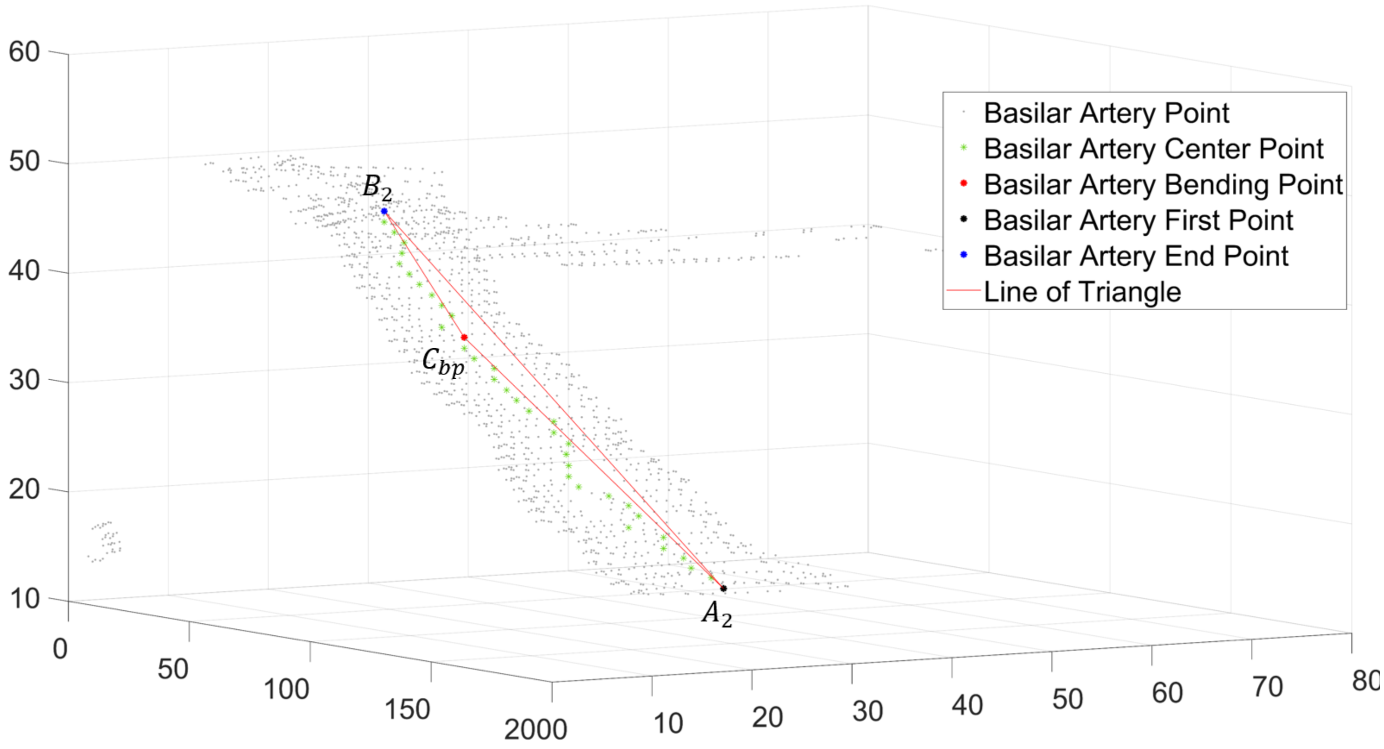
Basilar Artery Point
Basilar Artery Center Point
Basilar Artery Bending Point
Basilar Artery First Point
Basilar Artery End Point
Line of Triangle
B_2
C_{bp}
A_2

(r)

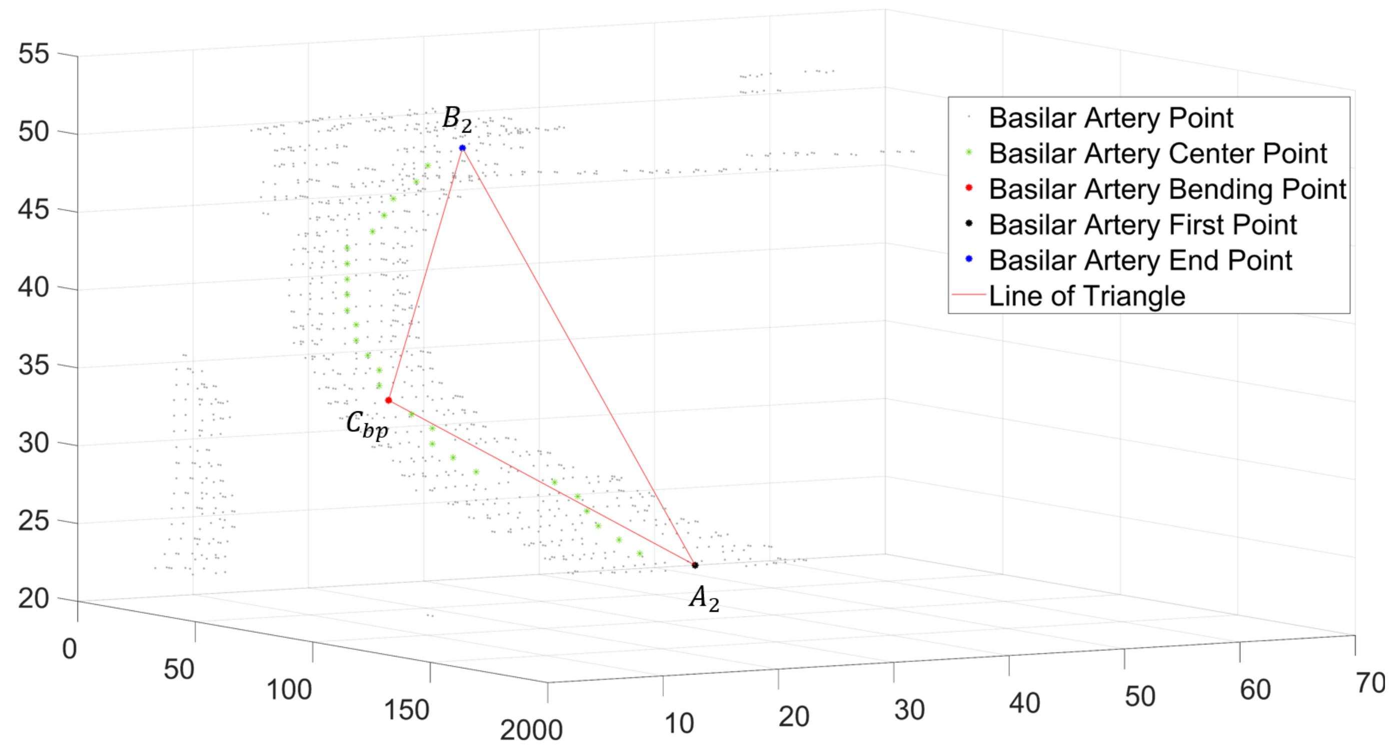

B_2
C_{bp}
A_2
Basilar Artery Point
Basilar Artery Center Point
Basilar Artery Bending Point
Basilar Artery First Point
Basilar Artery End Point
Line of Triangle
55
50
45
40
35
30
25
20
0
50
100
150
2000
10
20
30
40
50
60
70

(s)

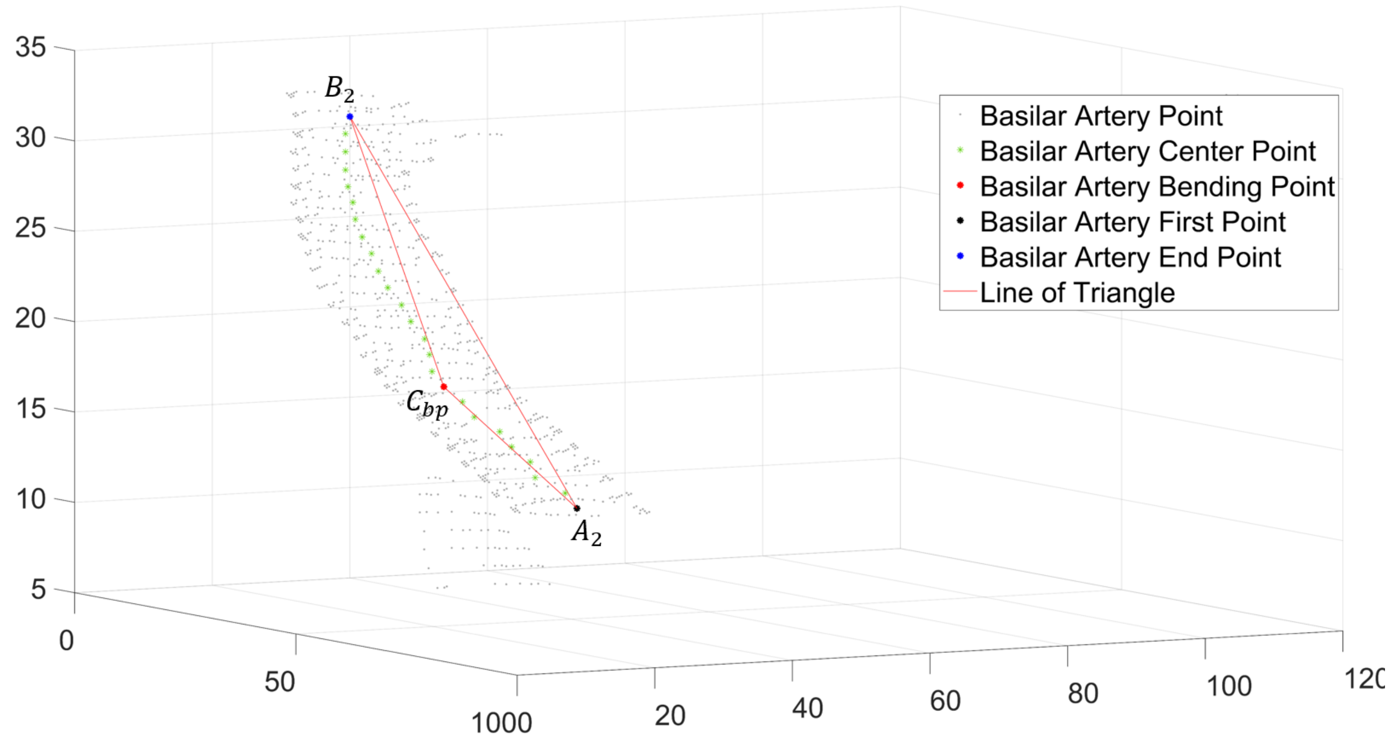

B_2
C_{bp}
A_2
Basilar Artery Point
Basilar Artery Center Point
Basilar Artery Bending Point
Basilar Artery First Point
Basilar Artery End Point
Line of Triangle
35
30
25
20
15
10
5
0
50
1000
20
40
60
80
100

(t)

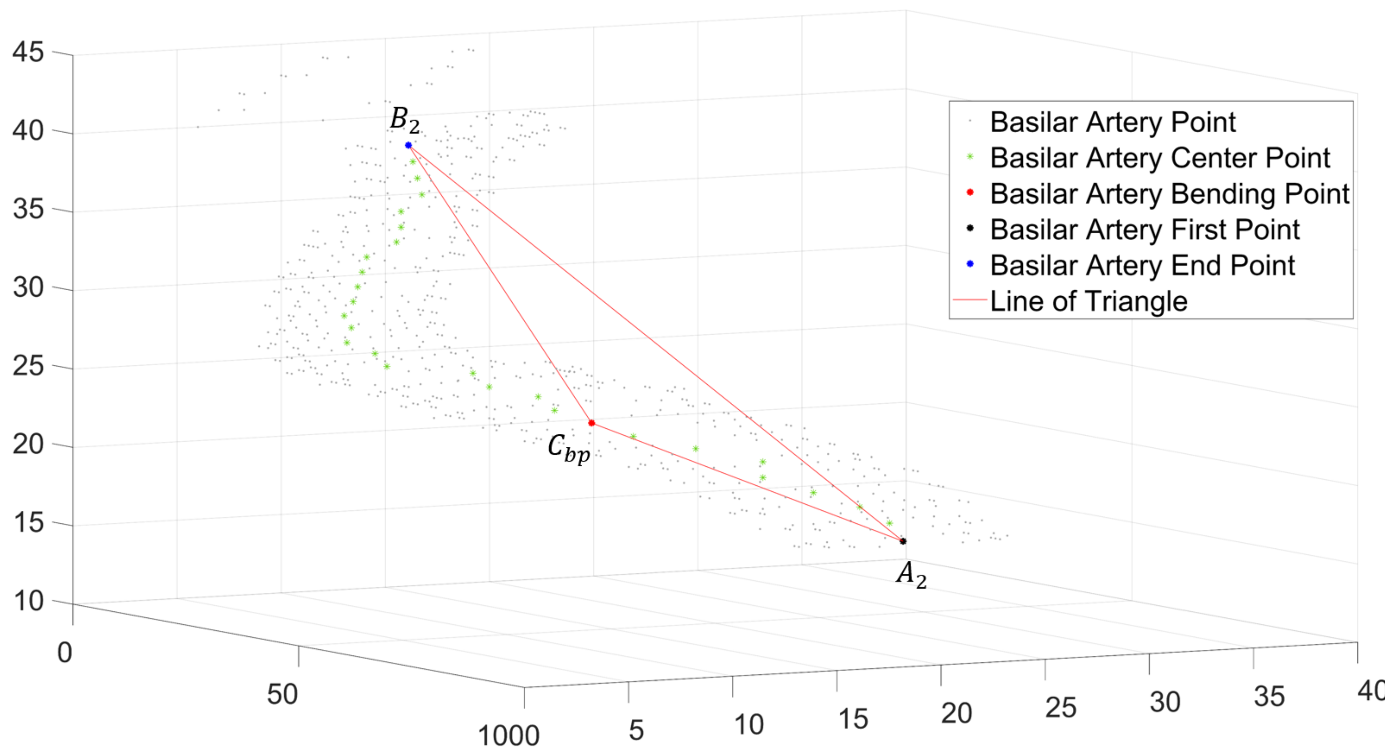
Basilar Artery Point
Basilar Artery Center Point
Basilar Artery Bending Point
Basilar Artery First Point
Basilar Artery End Point
Line of Triangle
B2
Cbp
A2
45
40
35
30
25
20
15
10
0
50
1000
5
10
15
20
25
30
35
40

(u)

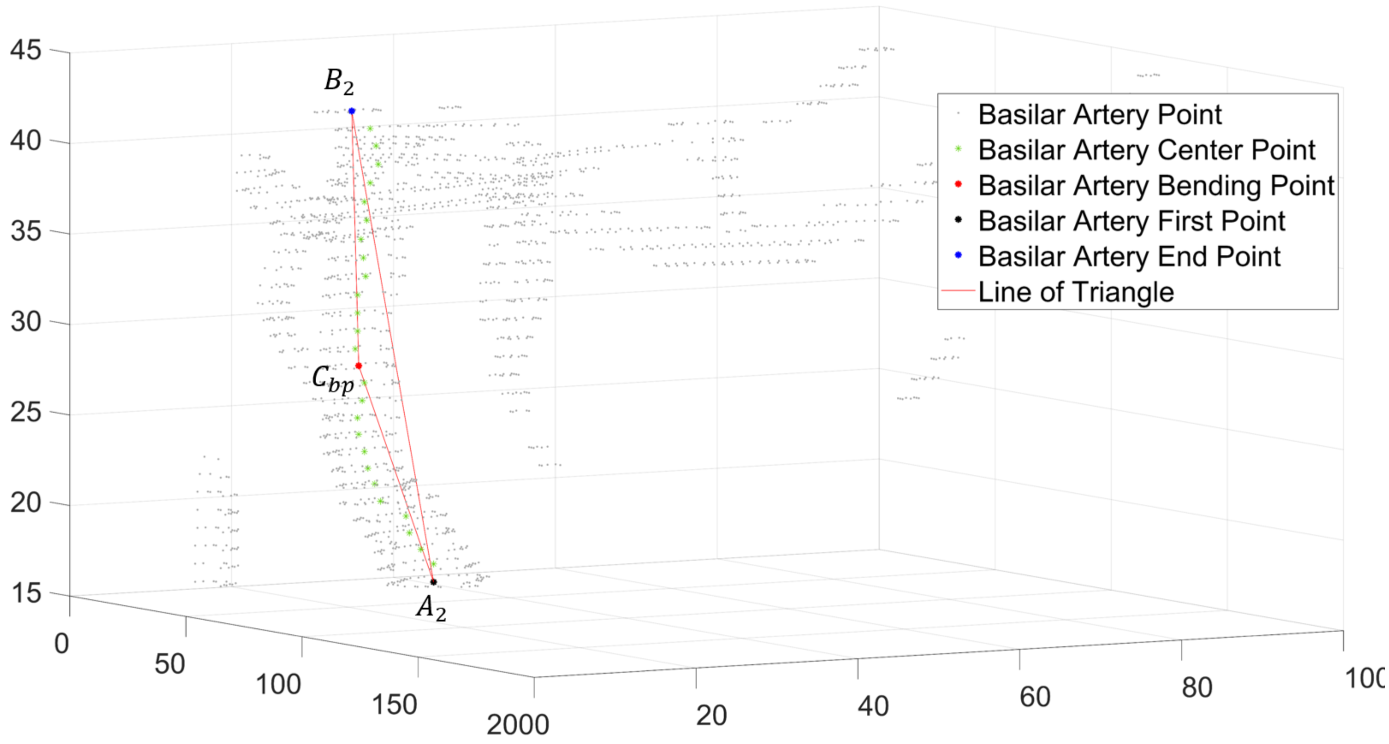
Basilar Artery Point
Basilar Artery Center Point
Basilar Artery Bending Point
Basilar Artery First Point
Basilar Artery End Point
Line of Triangle
B2
Cbp
A2
45
40
35
30
25
20
15
0
50
100
150
2000
20
40
60
80
100

(v)

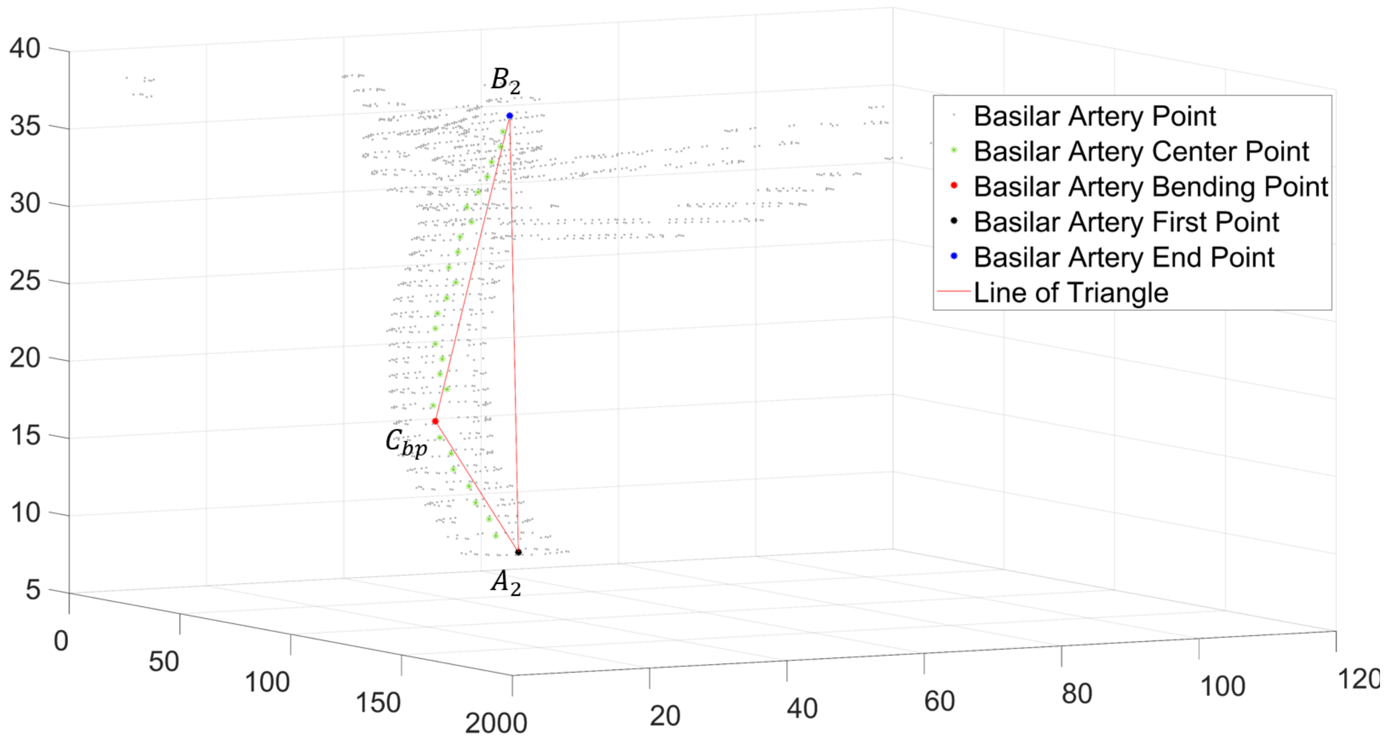
B_2
C_{bp}
A_2
Basilar Artery Point
Basilar Artery Center Point
Basilar Artery Bending Point
Basilar Artery First Point
Basilar Artery End Point
Line of Triangle

(w)

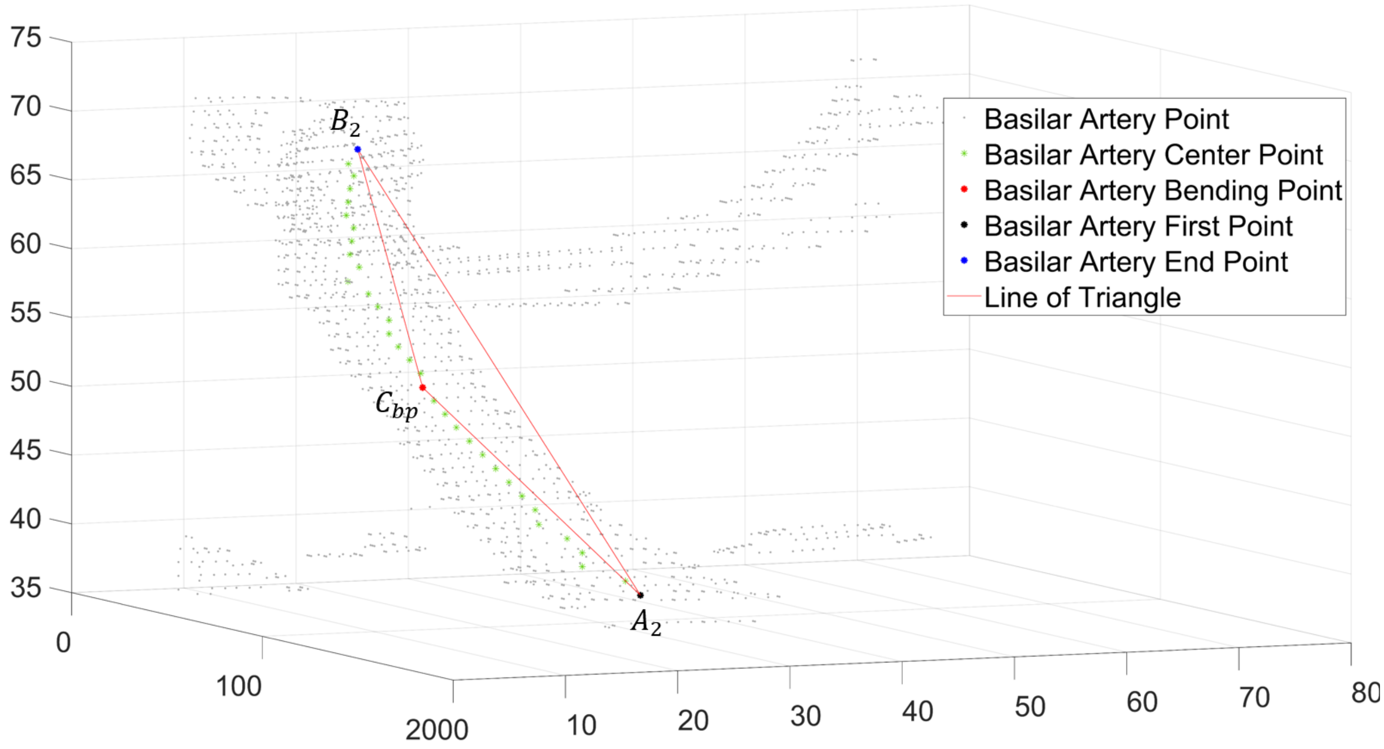
B_2
C_{bp}
A_2
Basilar Artery Point
Basilar Artery Center Point
Basilar Artery Bending Point
Basilar Artery First Point
Basilar Artery End Point
Line of Triangle

(x)

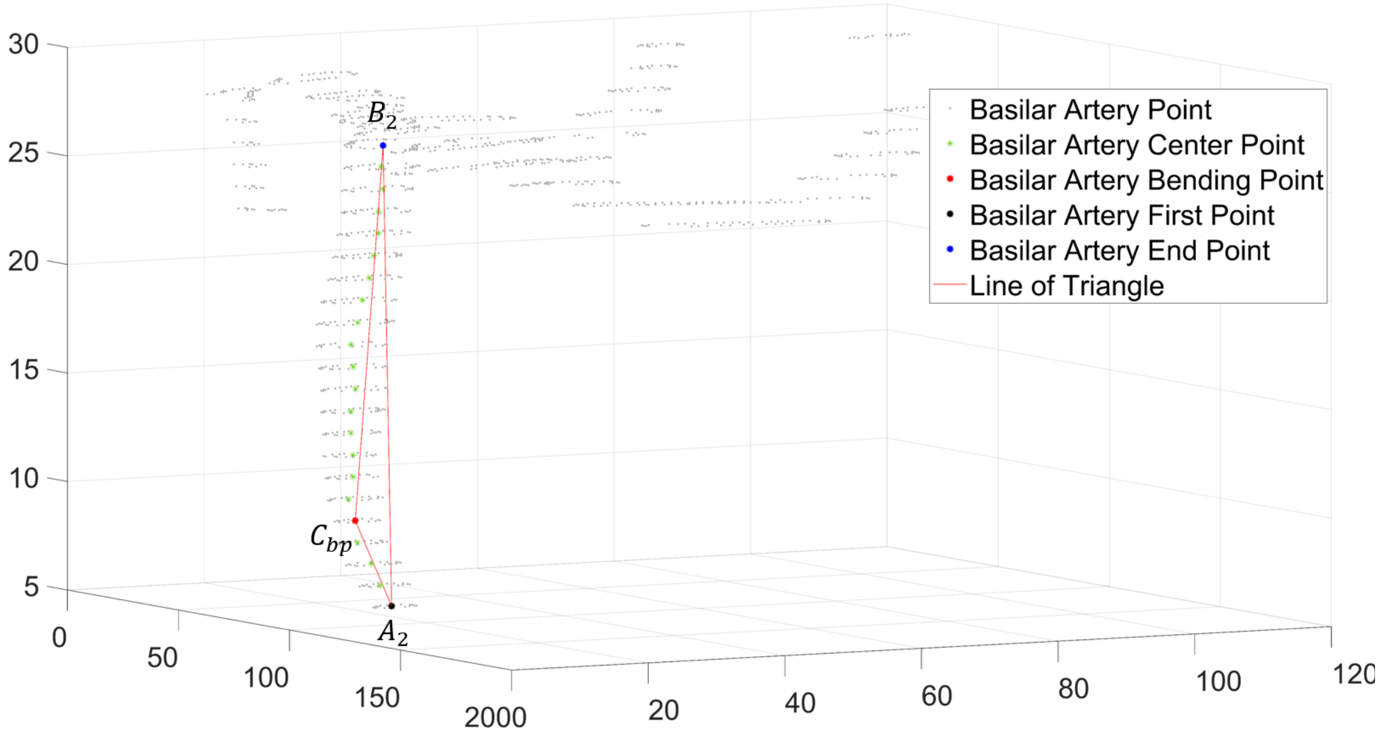

Basilar Artery Point
Basilar Artery Center Point
Basilar Artery Bending Point
Basilar Artery First Point
Basilar Artery End Point
Line of Triangle
B2
Cbp
A2
30
25
20
15
10
5
0
50
100
150
2000
20
40
60
80
100
120

(y)

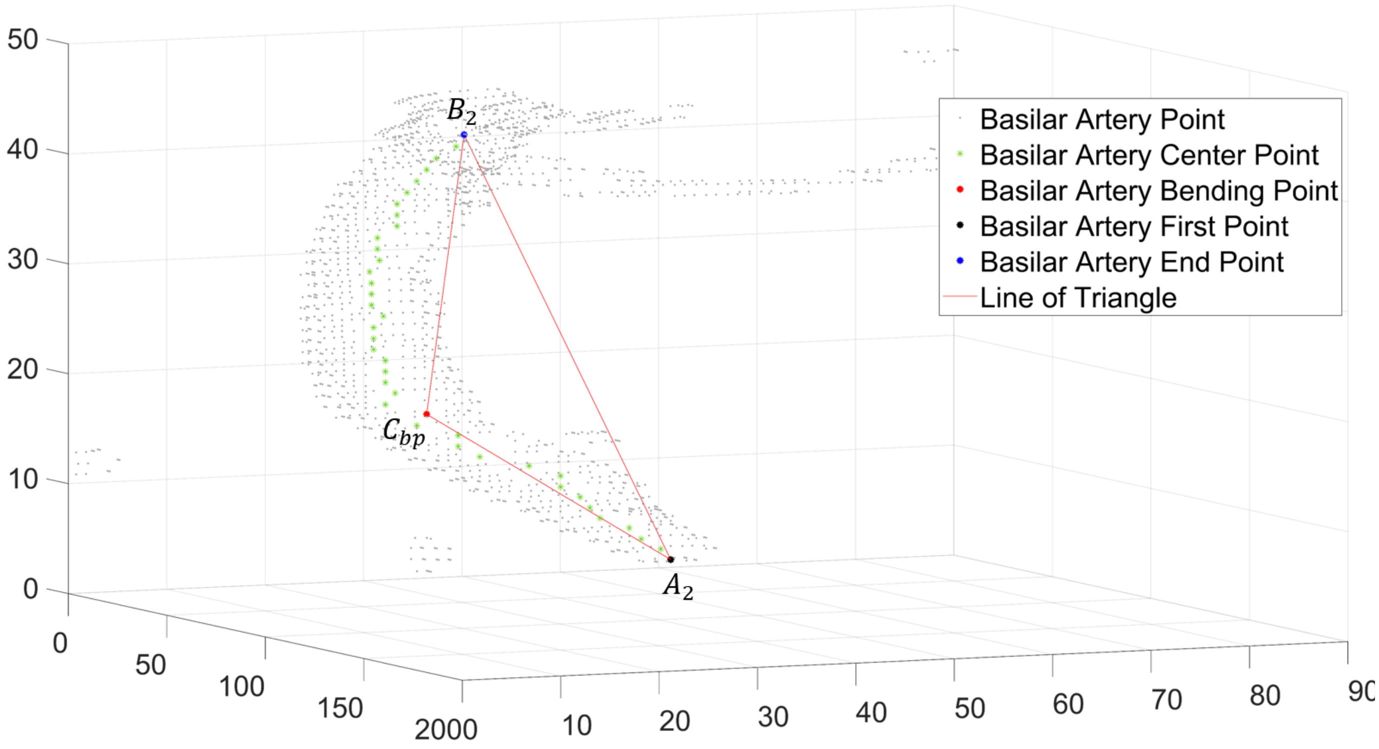

Basilar Artery Point
Basilar Artery Center Point
Basilar Artery Bending Point
Basilar Artery First Point
Basilar Artery End Point
Line of Triangle
B2
Cbp
A2
50
40
30
20
10
0
0
50
100
150
2000
10
20
30
40
50
60
70
80
90

(z)

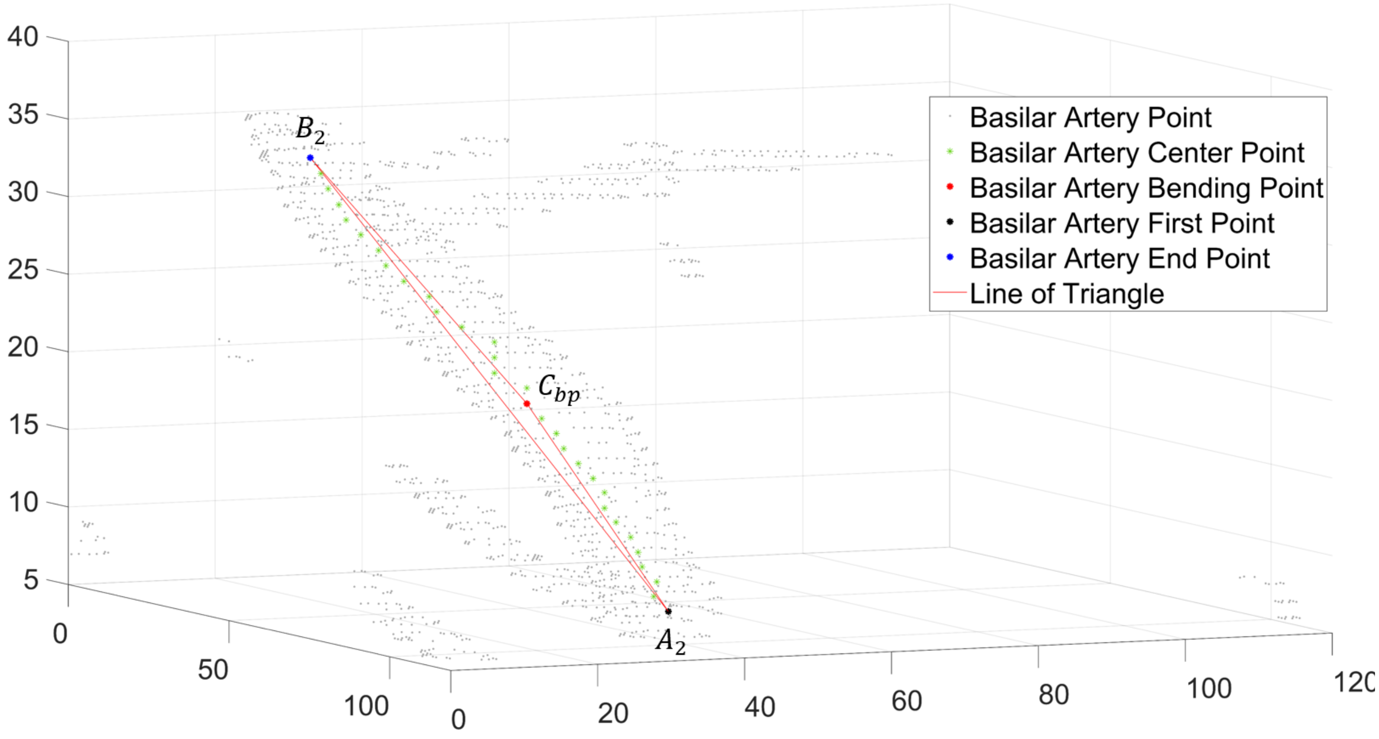

B_2
C_{bp}
A_2
Basilar Artery Point
Basilar Artery Center Point
Basilar Artery Bending Point
Basilar Artery First Point
Basilar Artery End Point
Line of Triangle
40
35
30
25
20
15
10
5
0
50
100
0
20
40
60
80
100
120

(aa)

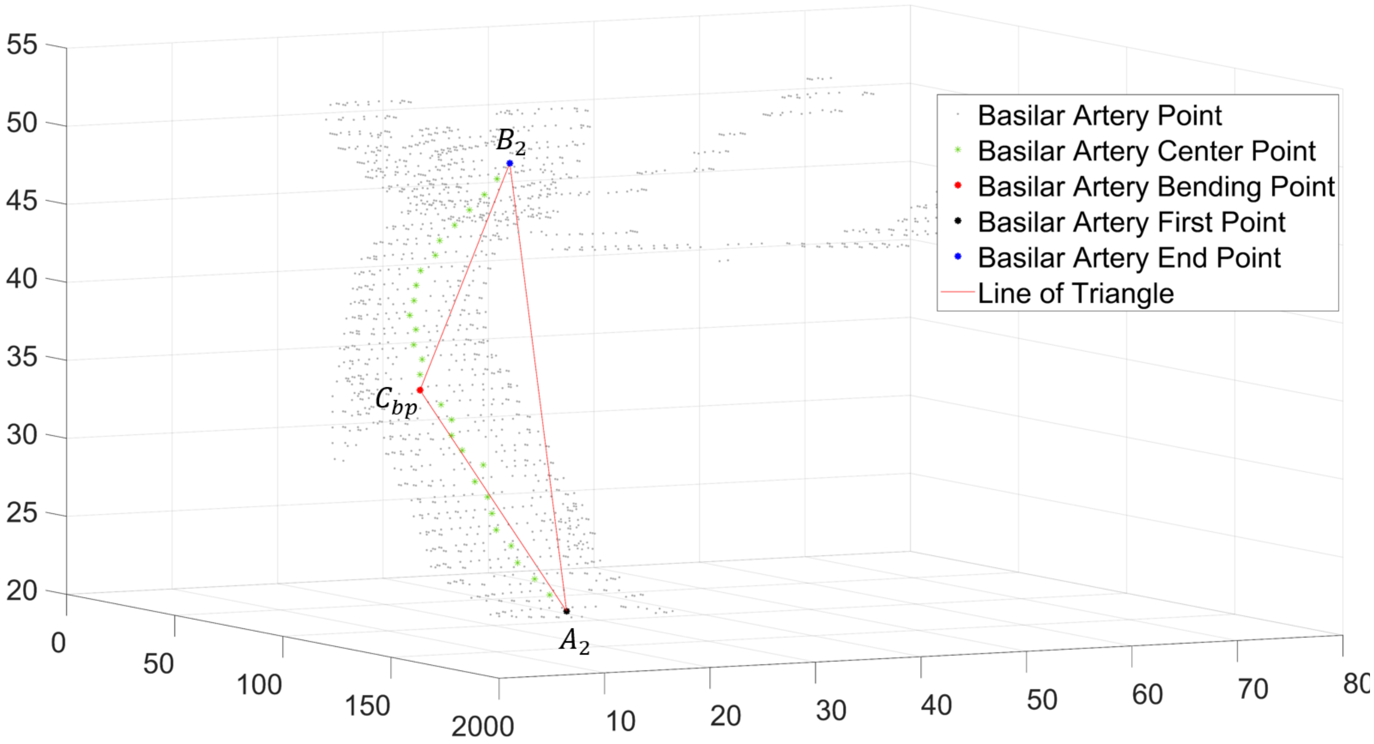

B_2
C_{bp}
A_2
Basilar Artery Point
Basilar Artery Center Point
Basilar Artery Bending Point
Basilar Artery First Point
Basilar Artery End Point
Line of Triangle
55
50
45
40
35
30
25
20
0
50
100
150
2000
10
20
30
40
50
60
70
80

(ab)

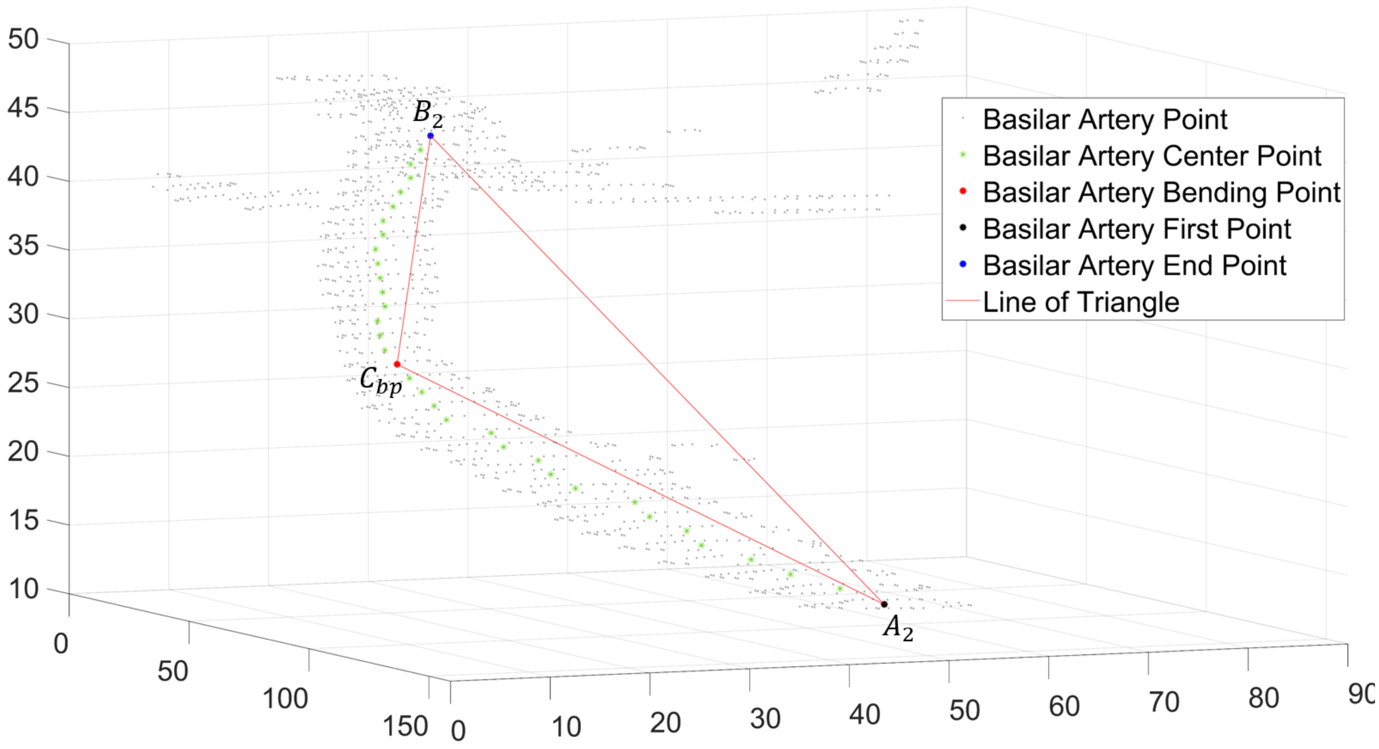
Basilar Artery Point
Basilar Artery Center Point
Basilar Artery Bending Point
Basilar Artery First Point
Basilar Artery End Point
Line of Triangle
B_2
C_{bp}
A_2

(ac)

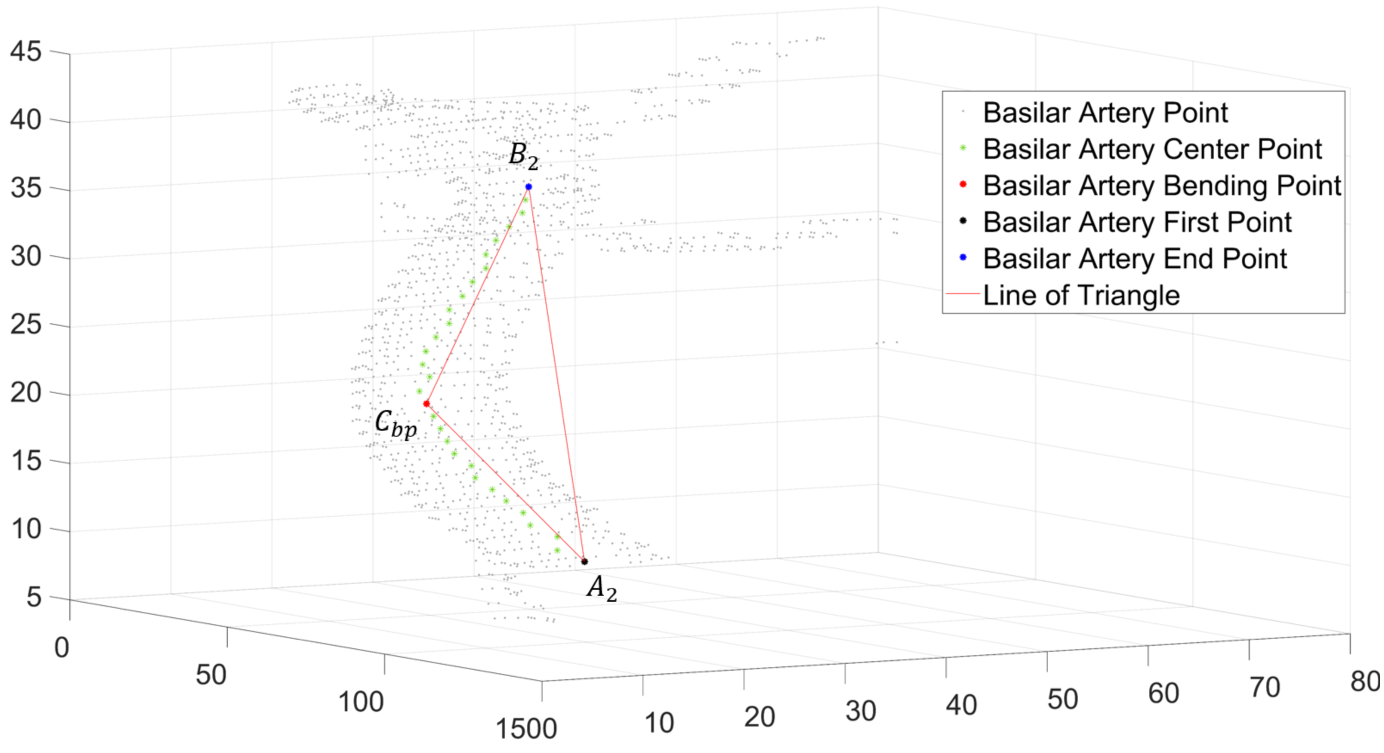
Basilar Artery Point
Basilar Artery Center Point
Basilar Artery Bending Point
Basilar Artery First Point
Basilar Artery End Point
Line of Triangle
B_2
C_{bp}
A_2

(ad)

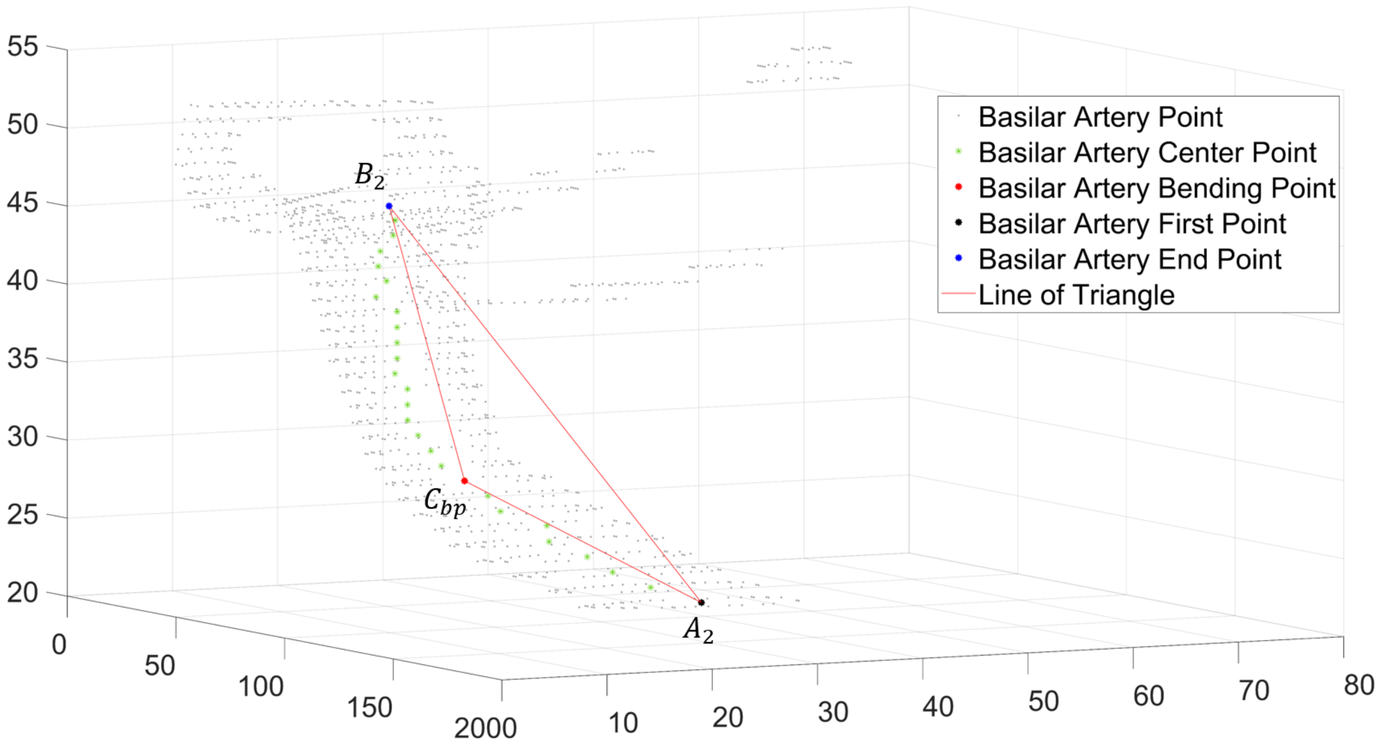

55
50
45
40
35
30
25
20
0
50
100
150
2000
10
20
30
40
50
60
70
80
B_2
C_{bp}
A_2
Basilar Artery Point
Basilar Artery Center Point
Basilar Artery Bending Point
Basilar Artery First Point
Basilar Artery End Point
Line of Triangle

(ae)

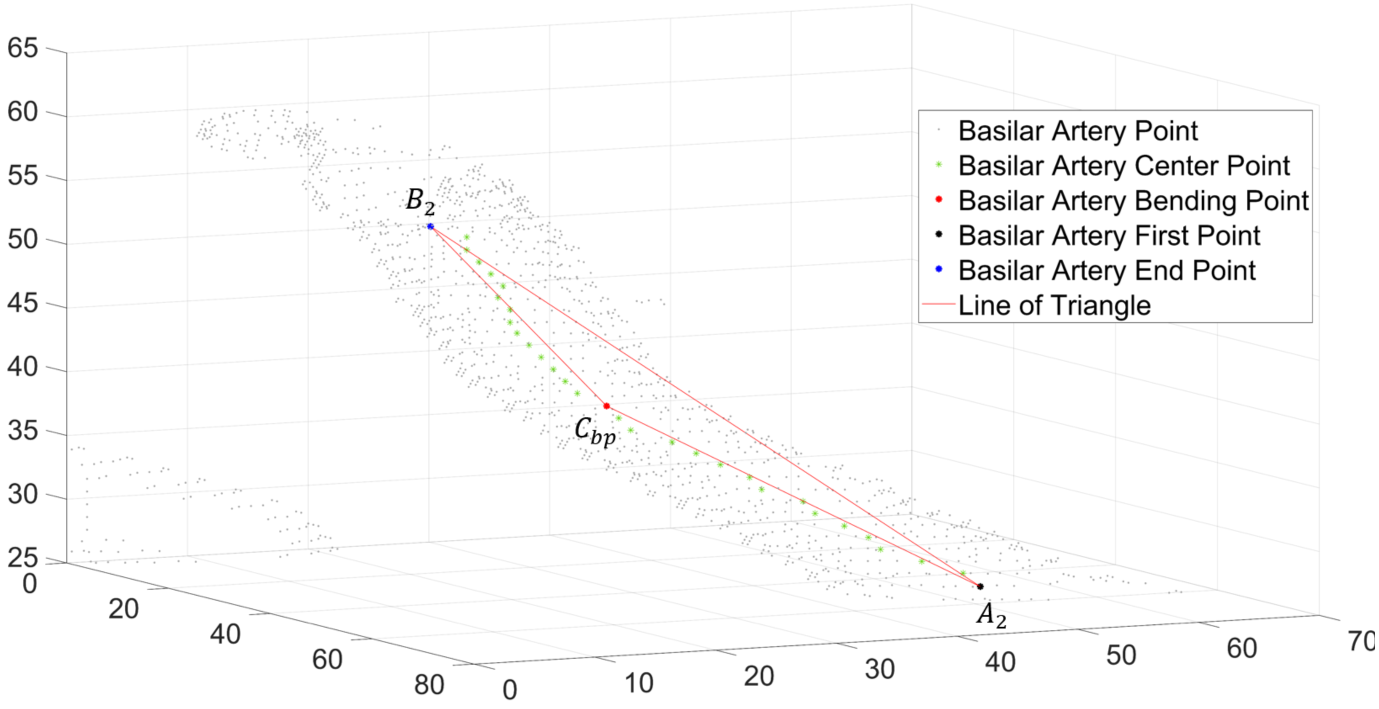

65
60
55
50
45
40
35
30
25
0
20
40
60
80
0
10
20
30
40
50
60
70
B_2
C_{bp}
A_2
Basilar Artery Point
Basilar Artery Center Point
Basilar Artery Bending Point
Basilar Artery First Point
Basilar Artery End Point
Line of Triangle

(af)

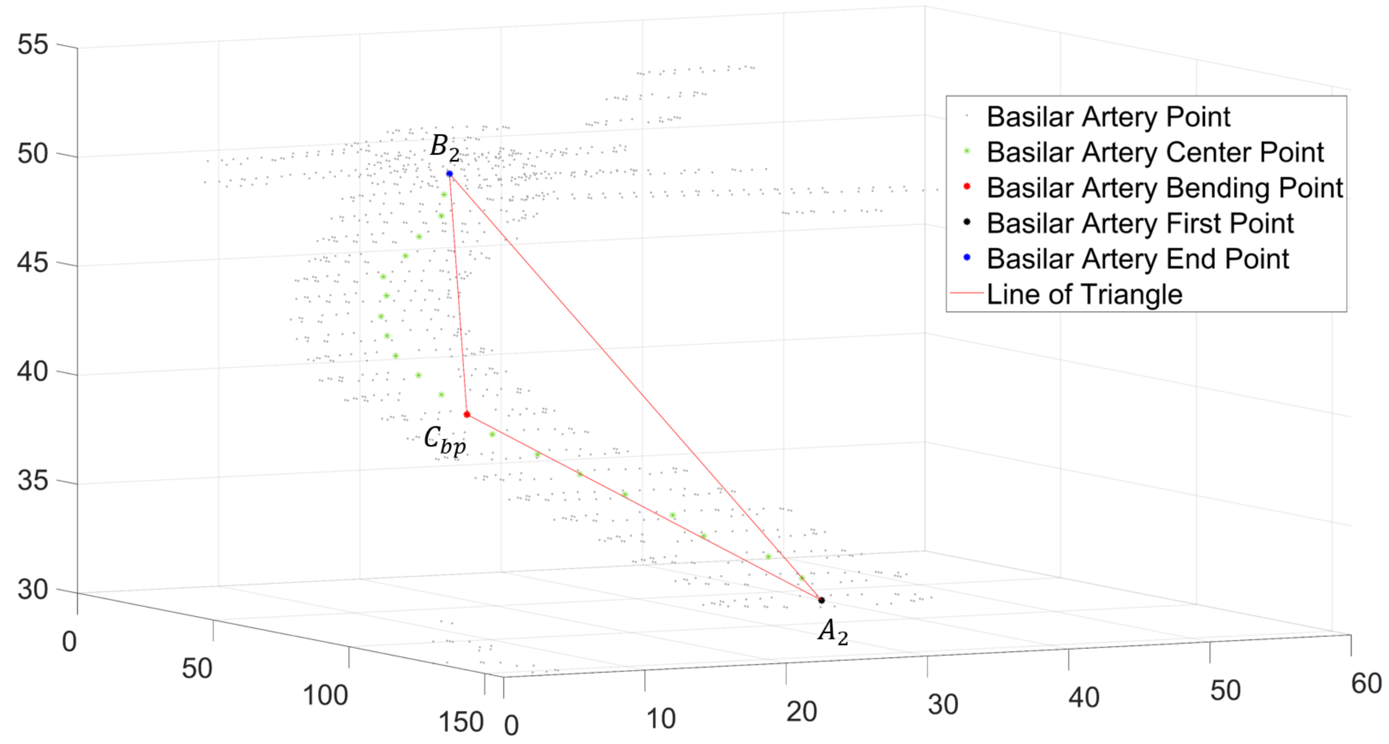

55
50
45
40
35
30
0
50
100
150
0
10
20
30
40
50
60
B_2
C_{bp}
A_2
Basilar Artery Point
Basilar Artery Center Point
Basilar Artery Bending Point
Basilar Artery First Point
Basilar Artery End Point
Line of Triangle

(ag)

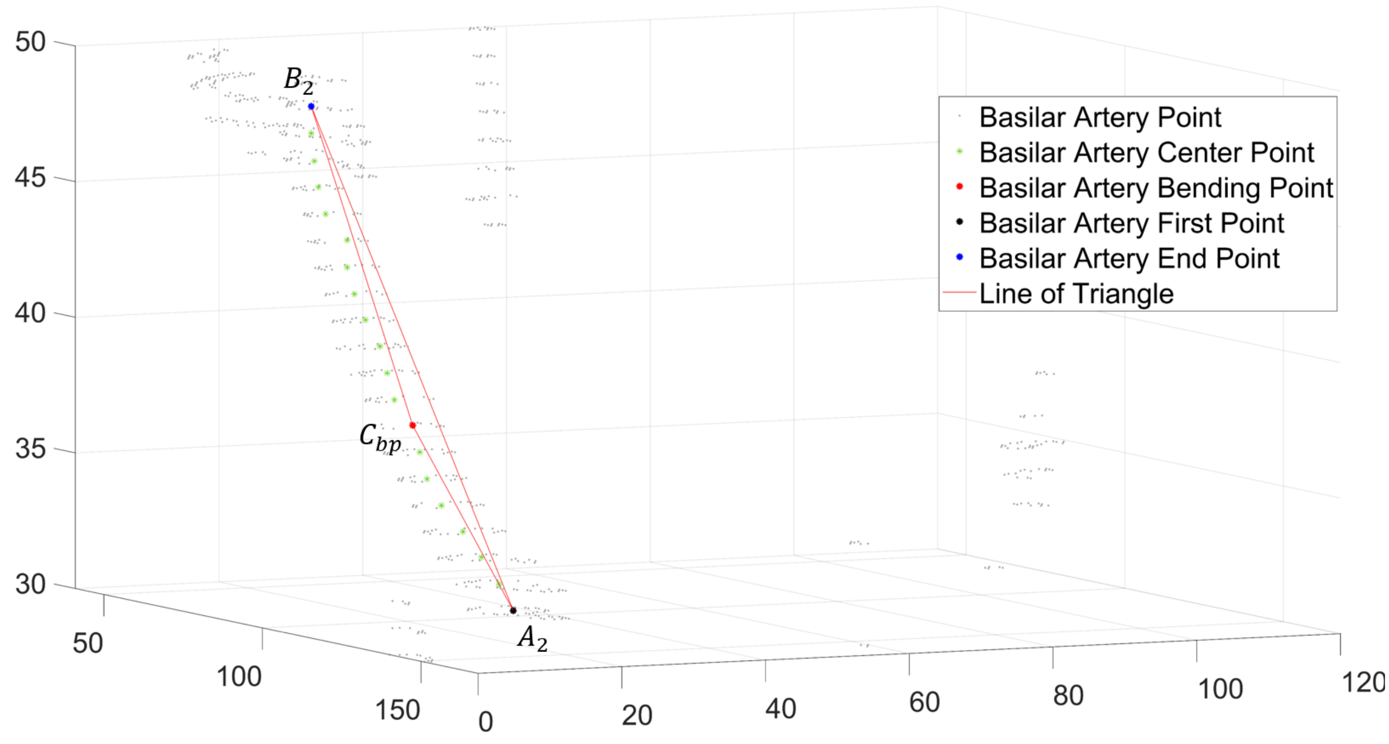

50
45
40
35
30
50
100
150
0
20
40
60
80
100
120
B_2
C_{bp}
A_2
Basilar Artery Point
Basilar Artery Center Point
Basilar Artery Bending Point
Basilar Artery First Point
Basilar Artery End Point
Line of Triangle

(ah)

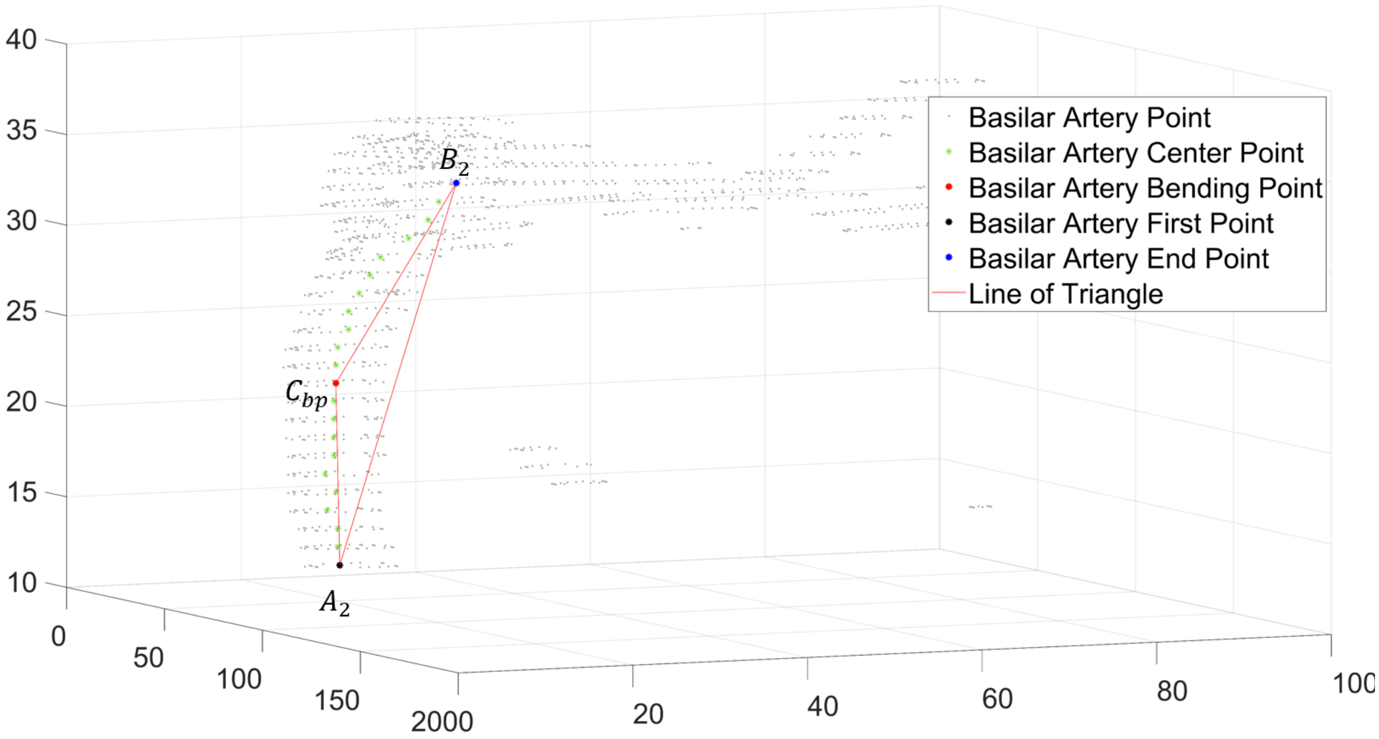

Basilar Artery Point
Basilar Artery Center Point
Basilar Artery Bending Point
Basilar Artery First Point
Basilar Artery End Point
Line of Triangle
B_2
C_{bp}
A_2
40
35
30
25
20
15
10
0
50
100
150
2000
20
40
60
80
100

(ai)

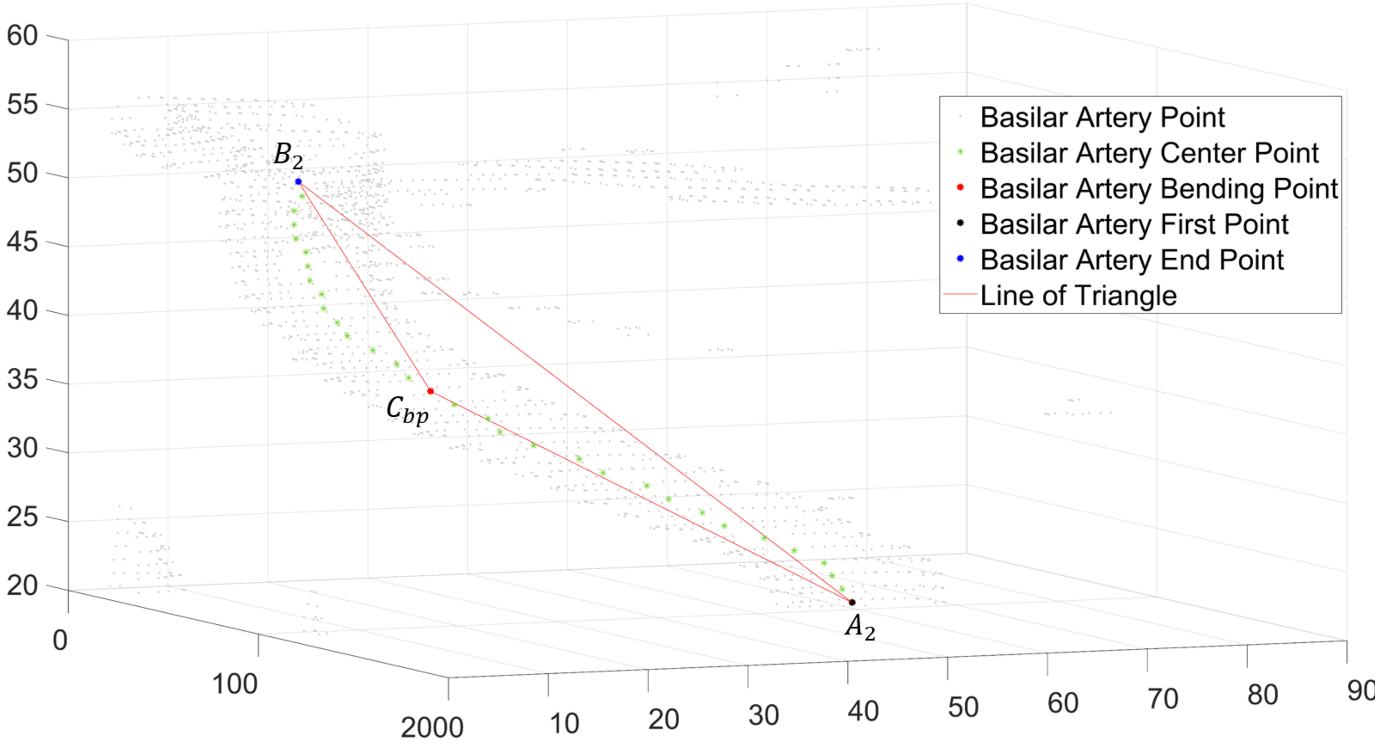

Basilar Artery Point
Basilar Artery Center Point
Basilar Artery Bending Point
Basilar Artery First Point
Basilar Artery End Point
Line of Triangle
B_2
C_{bp}
A_2
60
55
50
45
40
35
30
25
20
0
100
2000
10
20
30
40
50
60
70
80
90

(aj)

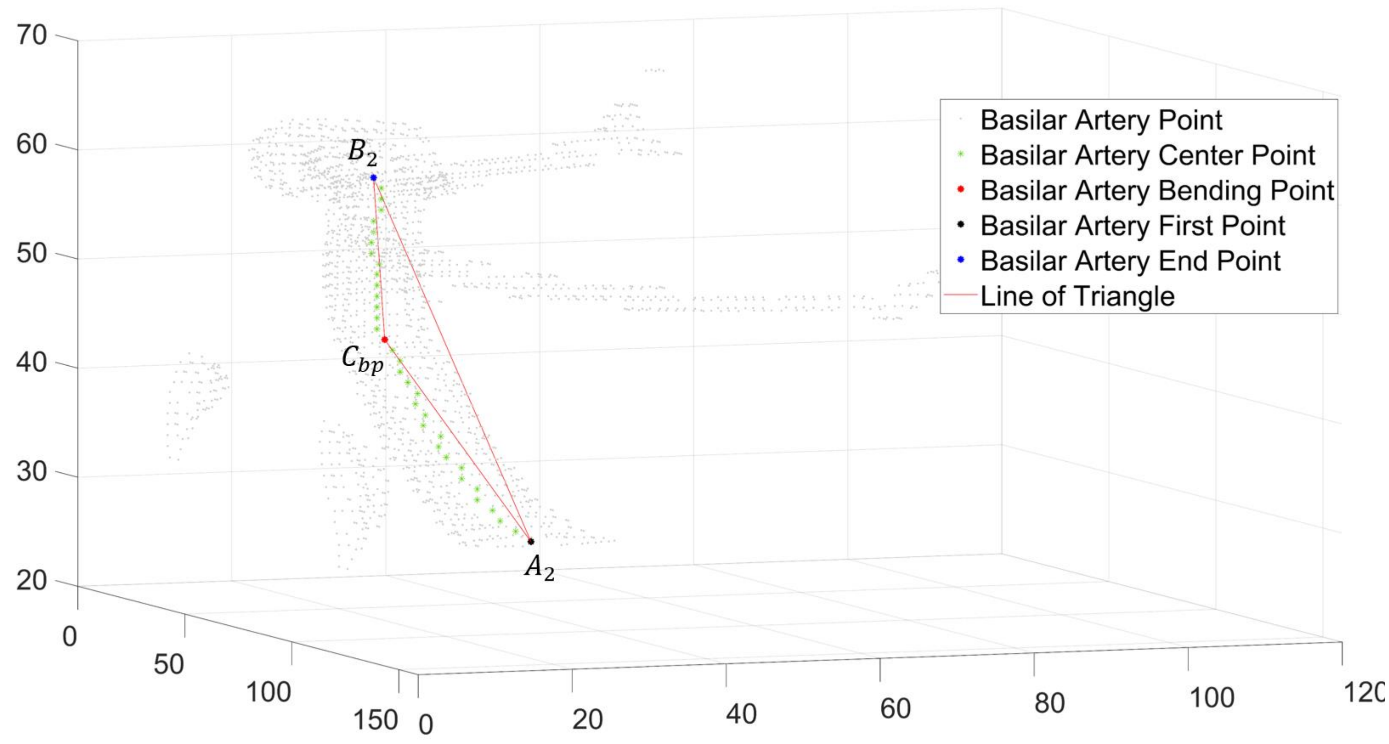

Basilar Artery Point
Basilar Artery Center Point
Basilar Artery Bending Point
Basilar Artery First Point
Basilar Artery End Point
Line of Triangle
B_2
C_{bp}
A_2
70
60
50
40
30
20
0
50
100
150
0
20
40
60
80
100
120

(ak)

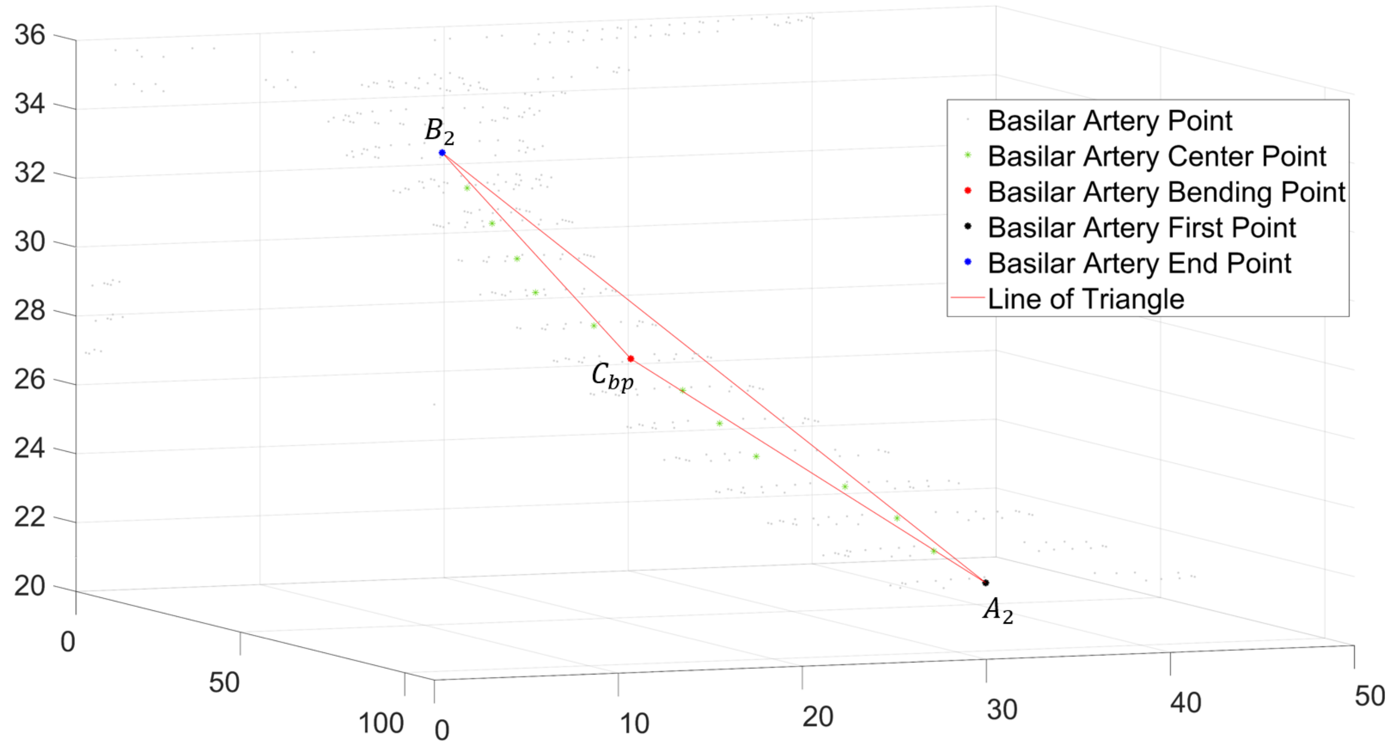

Basilar Artery Point
Basilar Artery Center Point
Basilar Artery Bending Point
Basilar Artery First Point
Basilar Artery End Point
Line of Triangle
B_2
C_{bp}
A_2
36
34
32
30
28
26
24
22
20
0
50
100
0
10
20
30
40
50

(al)

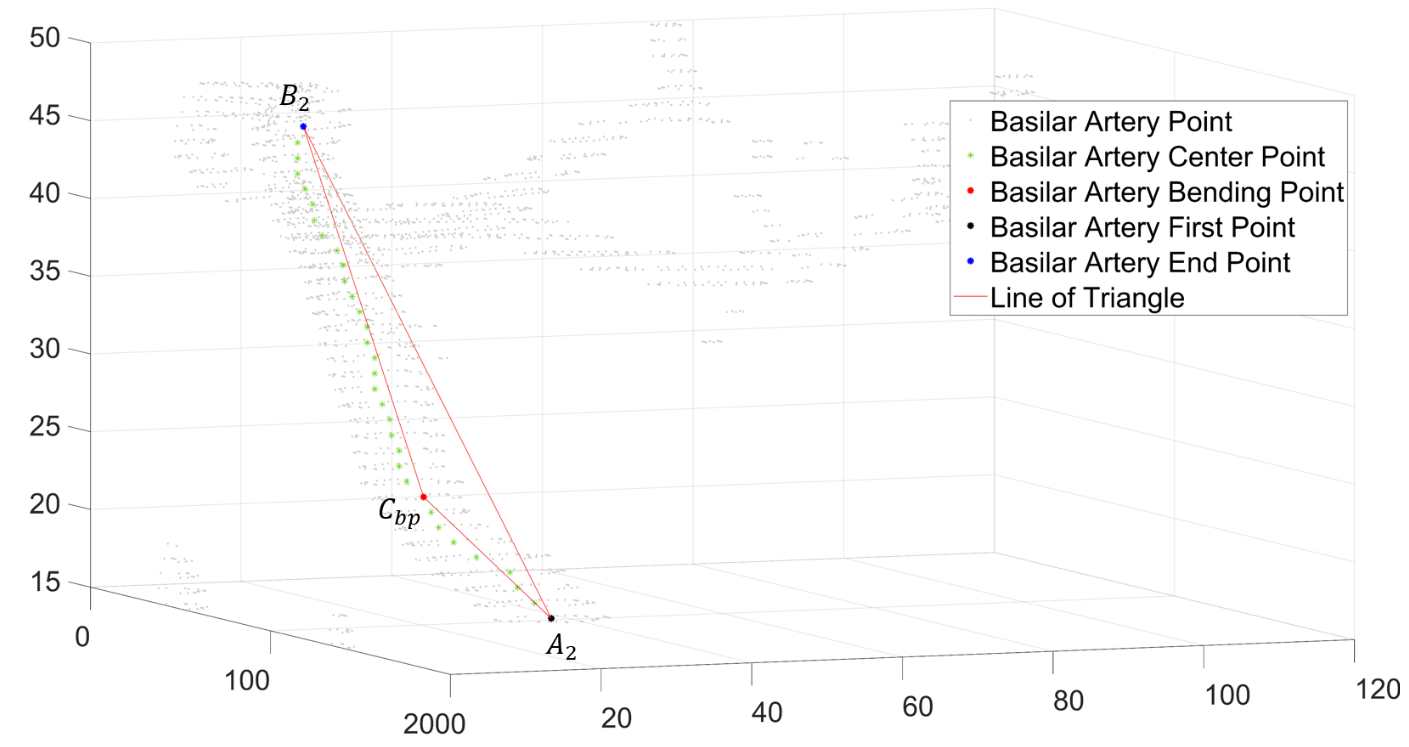

Basilar Artery Point
Basilar Artery Center Point
Basilar Artery Bending Point
Basilar Artery First Point
Basilar Artery End Point
Line of Triangle
B_2
C_{bp}
A_2
50
45
40
35
30
25
20
15
0
100
2000
20
40
60
80
100
120

(am)

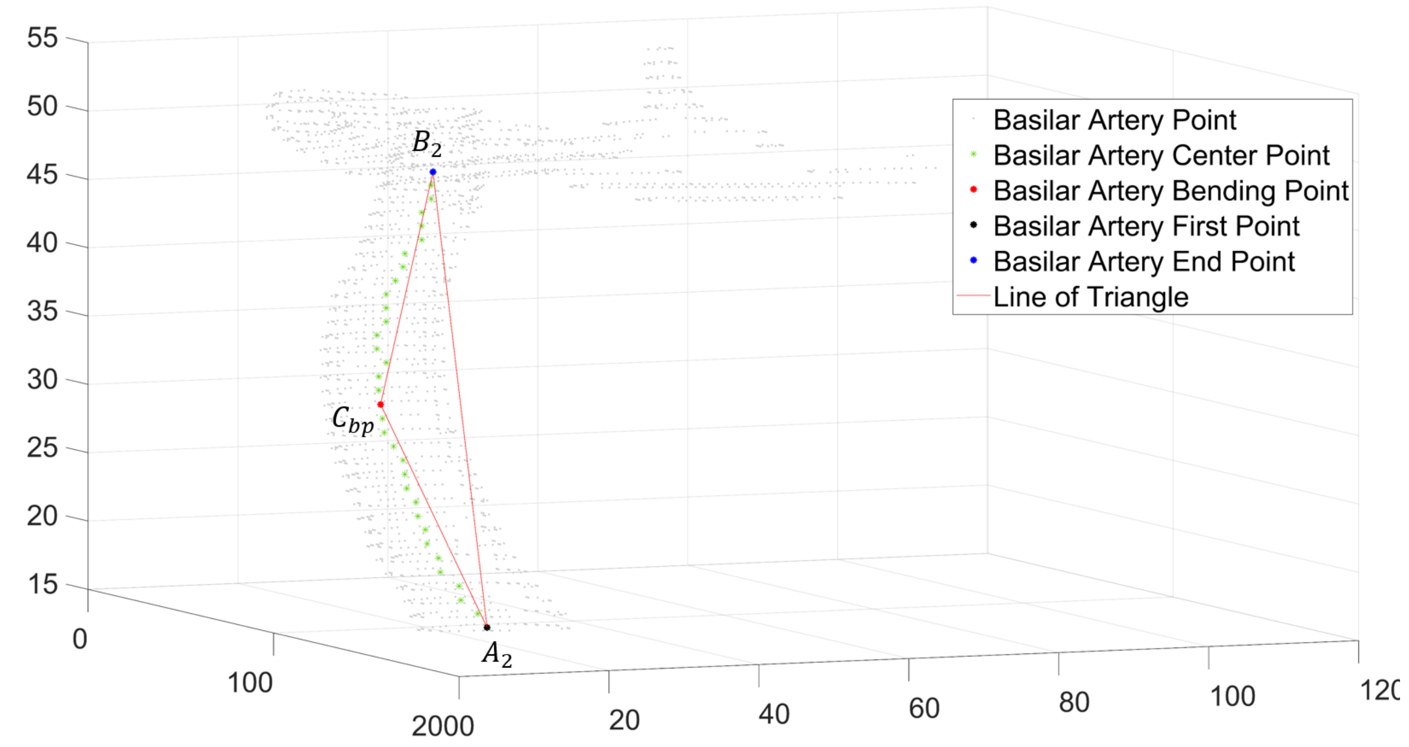

Basilar Artery Point
Basilar Artery Center Point
Basilar Artery Bending Point
Basilar Artery First Point
Basilar Artery End Point
Line of Triangle
B_2
C_{bp}
A_2
55
50
45
40
35
30
25
20
15
0
100
2000
20
40
60
80
100

(an)

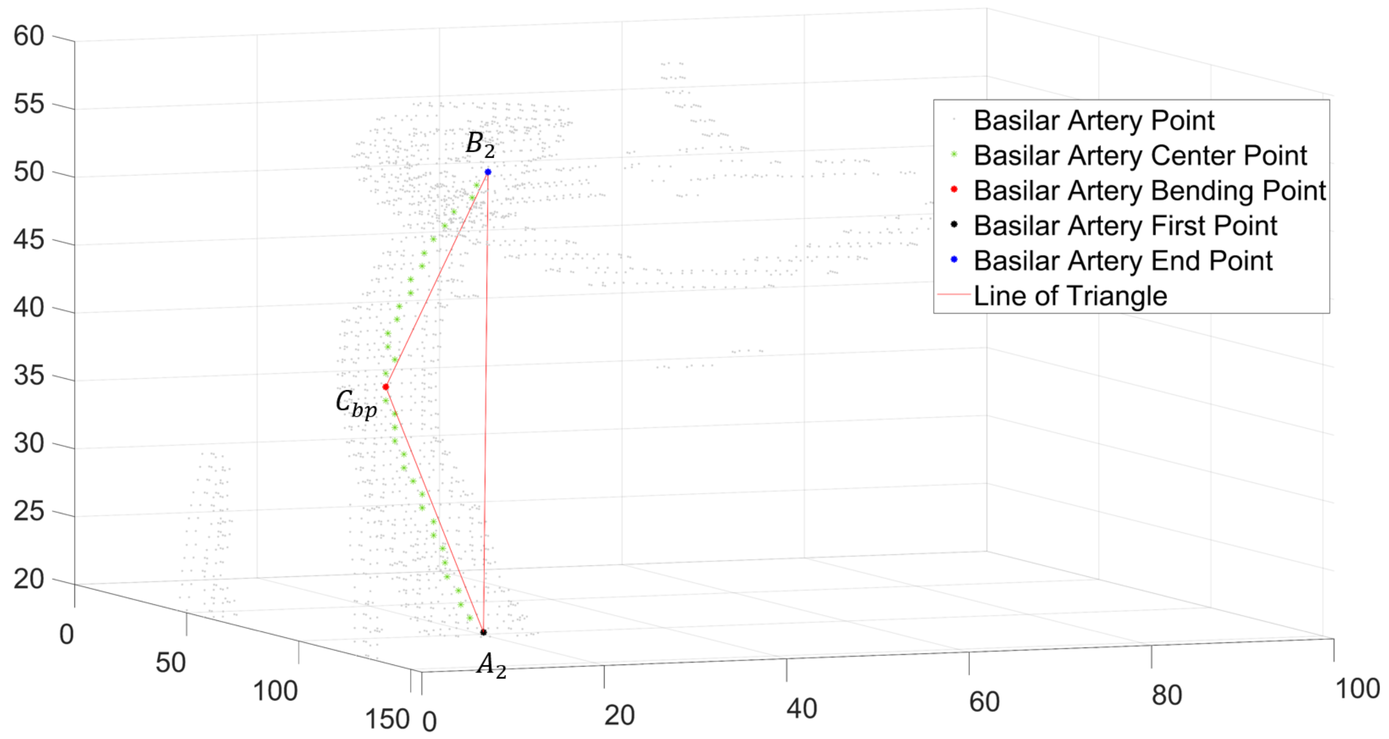

60
55
50
45
40
35
30
25
20
0
50
100
150
0
20
40
60
80
100
B_2
C_{bp}
A_2
Basilar Artery Point
Basilar Artery Center Point
Basilar Artery Bending Point
Basilar Artery First Point
Basilar Artery End Point
Line of Triangle

(ao)

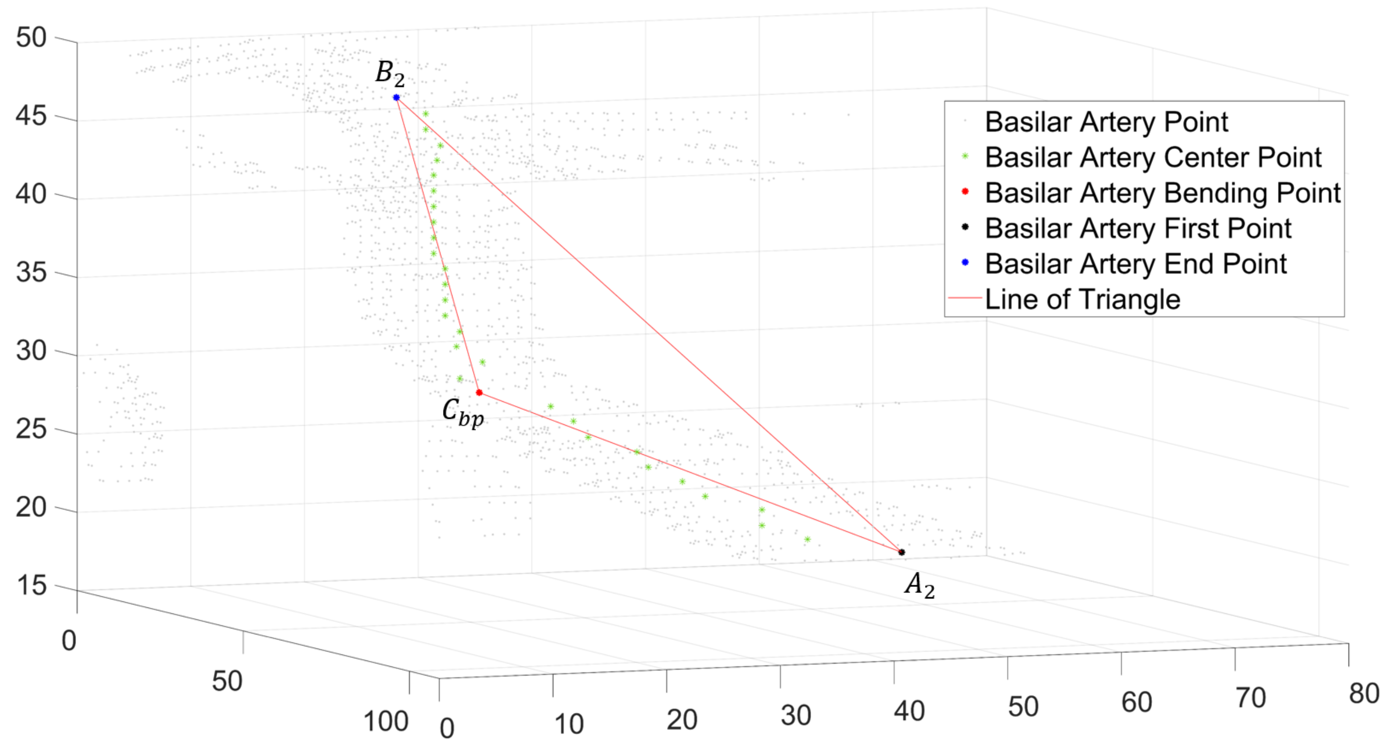

50
45
40
35
30
25
20
15
0
50
100
0
10
20
30
40
50
60
70
80
B_2
C_{bp}
A_2
Basilar Artery Point
Basilar Artery Center Point
Basilar Artery Bending Point
Basilar Artery First Point
Basilar Artery End Point
Line of Triangle

(ap)

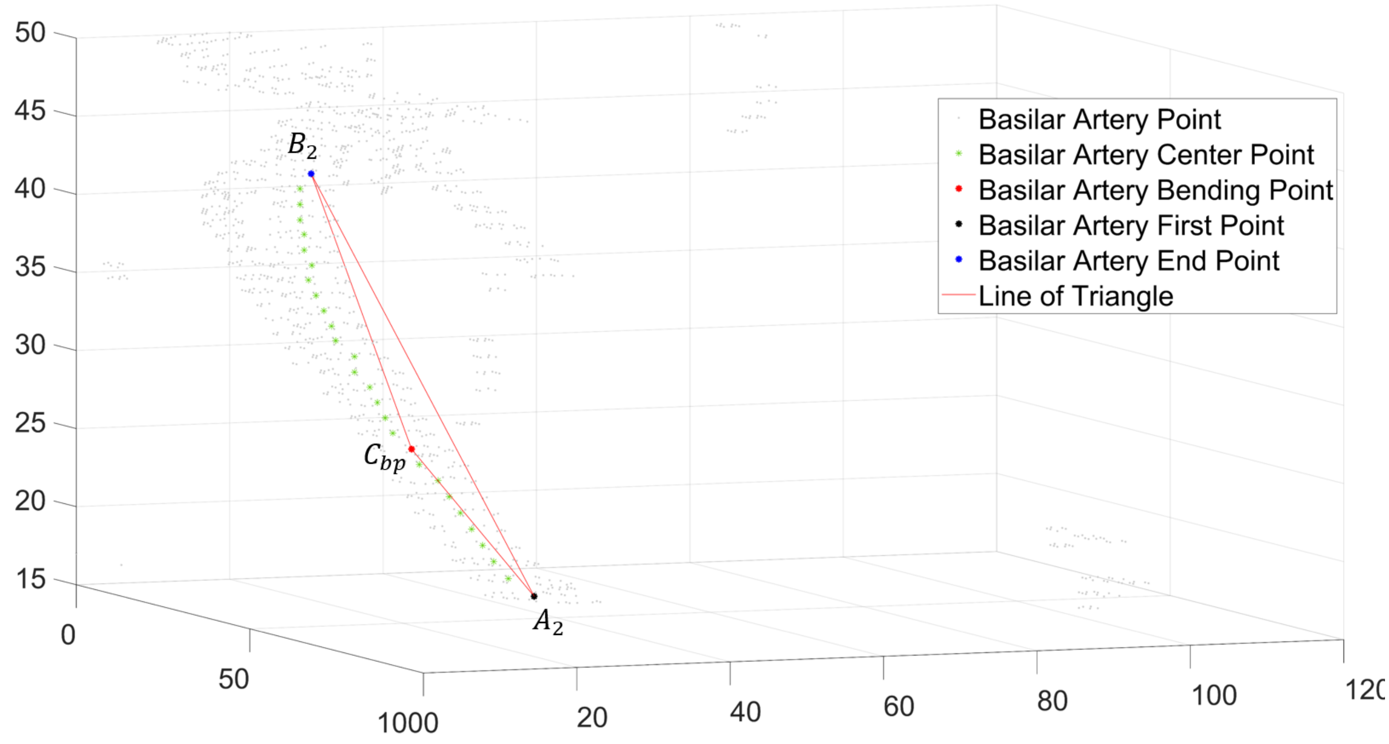
50
45
40
35
30
25
20
15
0
50
1000
20
40
60
80
100
B_2
C_{bp}
A_2
Basilar Artery Point
Basilar Artery Center Point
Basilar Artery Bending Point
Basilar Artery First Point
Basilar Artery End Point
Line of Triangle

(aq)

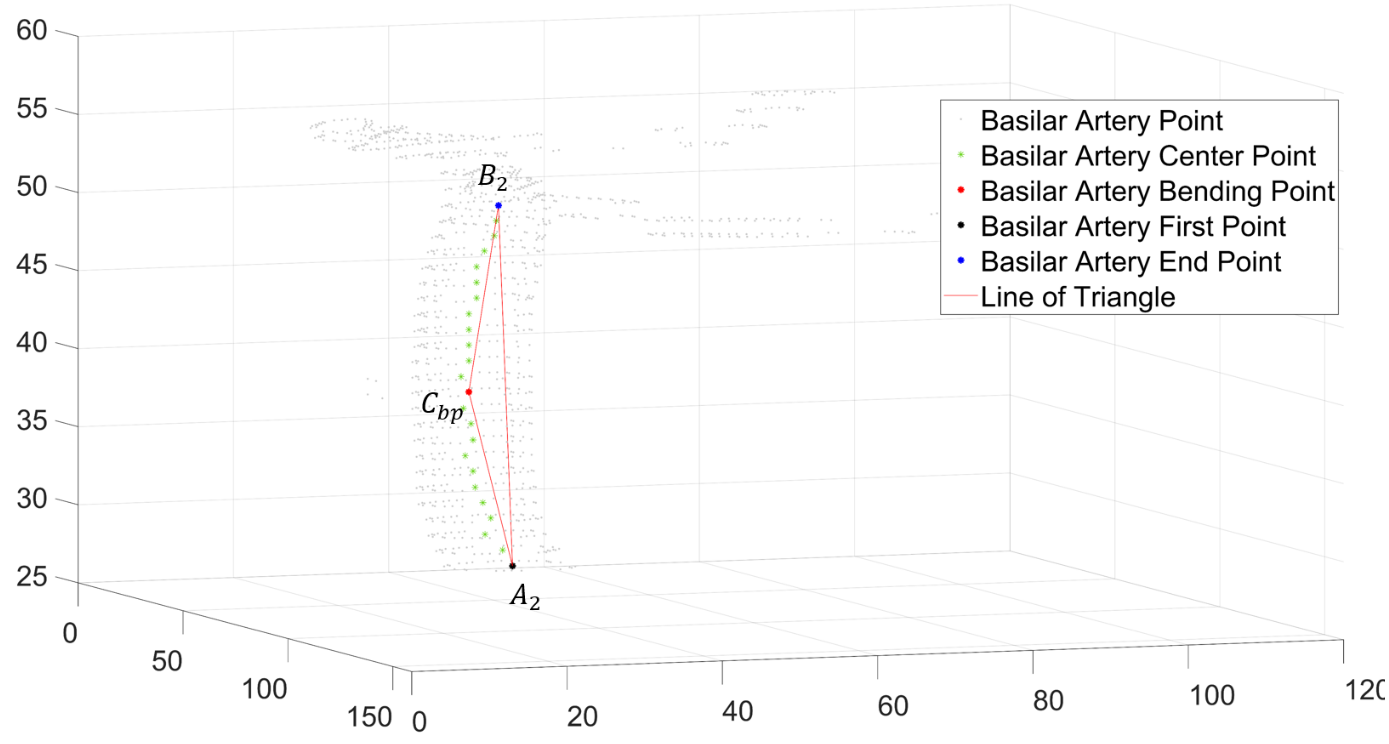
60
55
50
45
40
35
30
25
0
50
100
150
0
20
40
60
80
100
B_2
C_{bp}
A_2
Basilar Artery Point
Basilar Artery Center Point
Basilar Artery Bending Point
Basilar Artery First Point
Basilar Artery End Point
Line of Triangle

(ar)

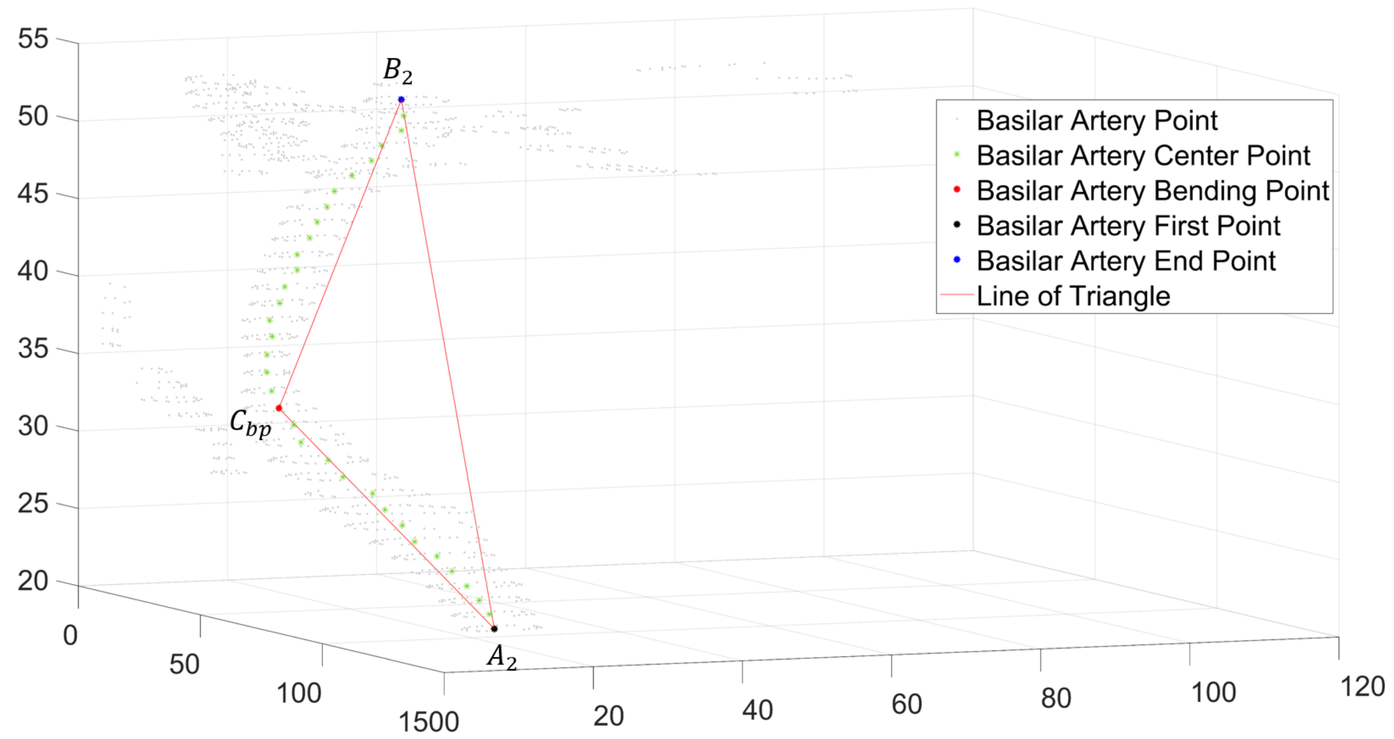
B_2
C_{bp}
A_2
Basilar Artery Point
Basilar Artery Center Point
Basilar Artery Bending Point
Basilar Artery First Point
Basilar Artery End Point
Line of Triangle
55
50
45
40
35
30
25
20
0
50
100
1500
20
40
60
80
100
120

(as)

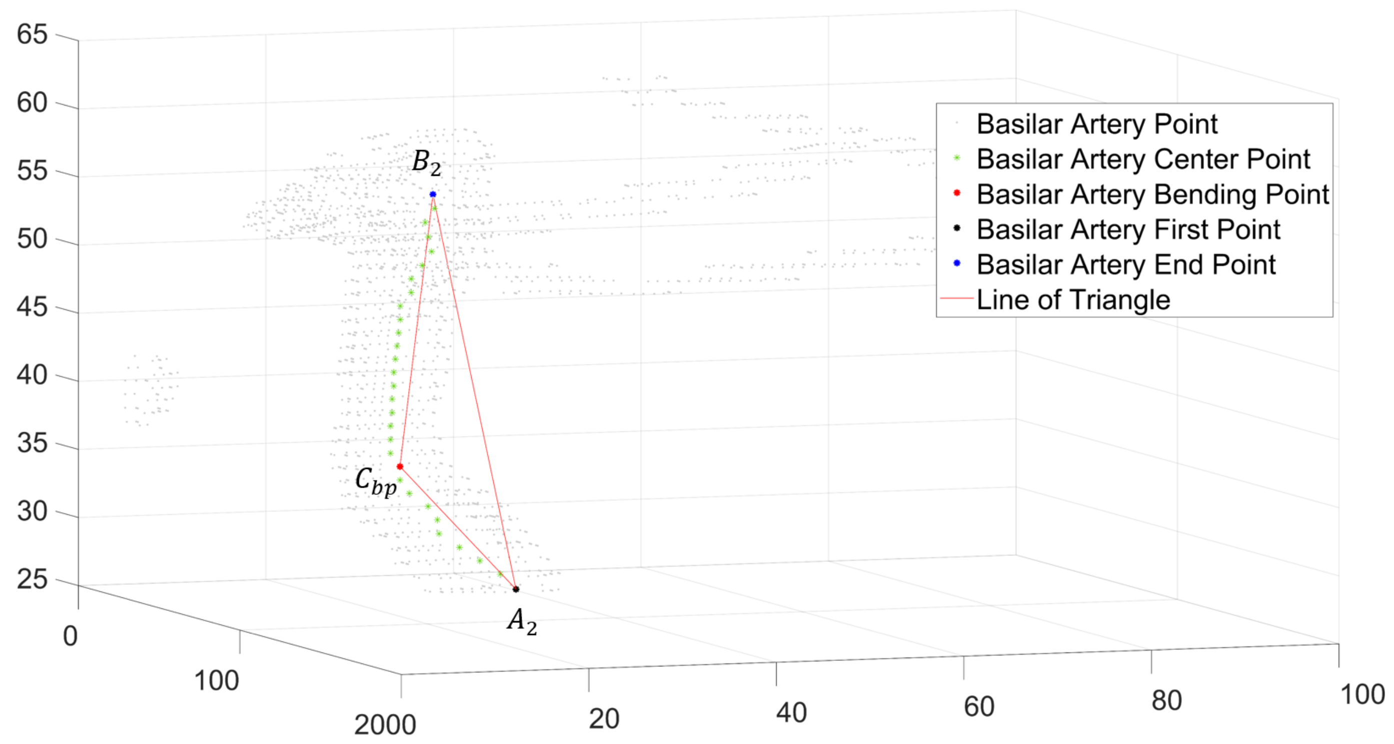
B_2
C_{bp}
A_2
Basilar Artery Point
Basilar Artery Center Point
Basilar Artery Bending Point
Basilar Artery First Point
Basilar Artery End Point
Line of Triangle
65
60
55
50
45
40
35
30
25
0
100
2000
20
40
60
80
100

(at)

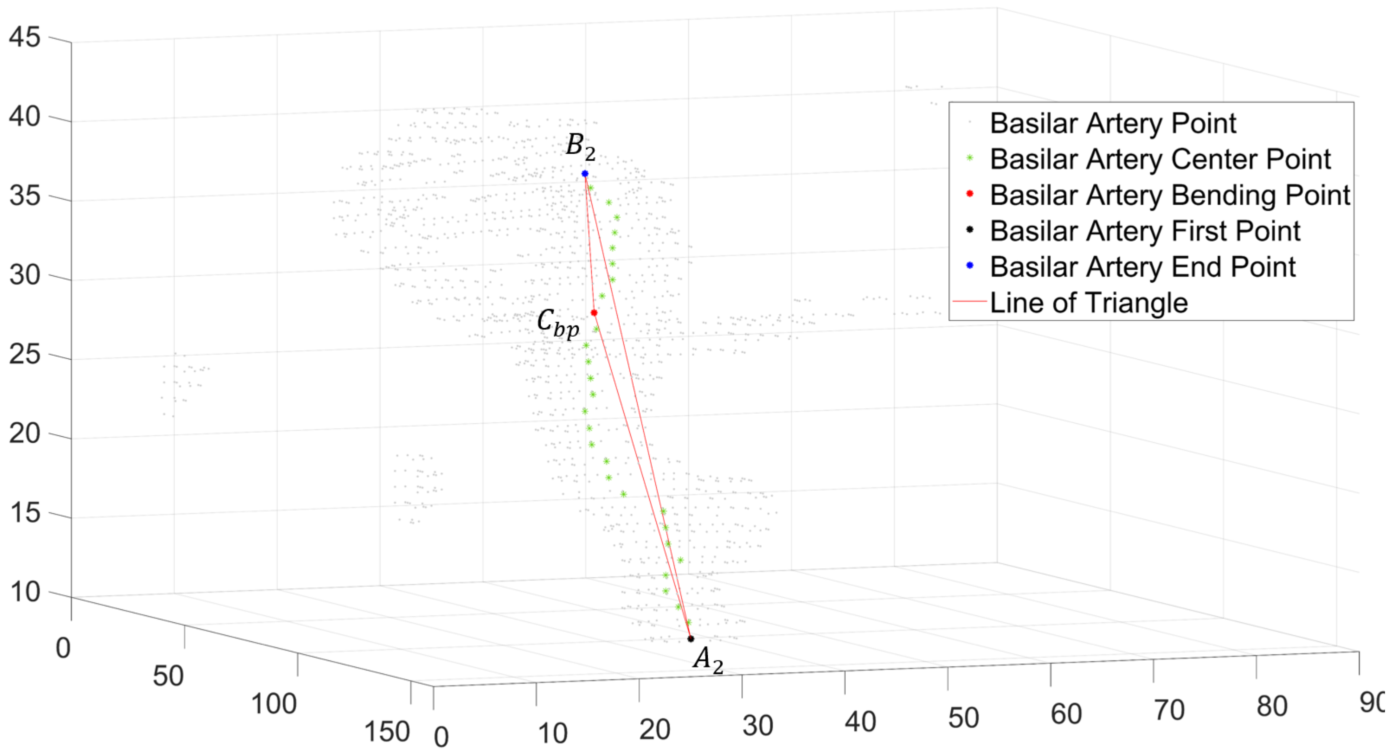

45
40
35
30
25
20
15
10
0
50
100
150
0
10
20
30
40
50
60
70
80
B_2
C_{bp}
A_2
Basilar Artery Point
Basilar Artery Center Point
Basilar Artery Bending Point
Basilar Artery First Point
Basilar Artery End Point
Line of Triangle

(au)

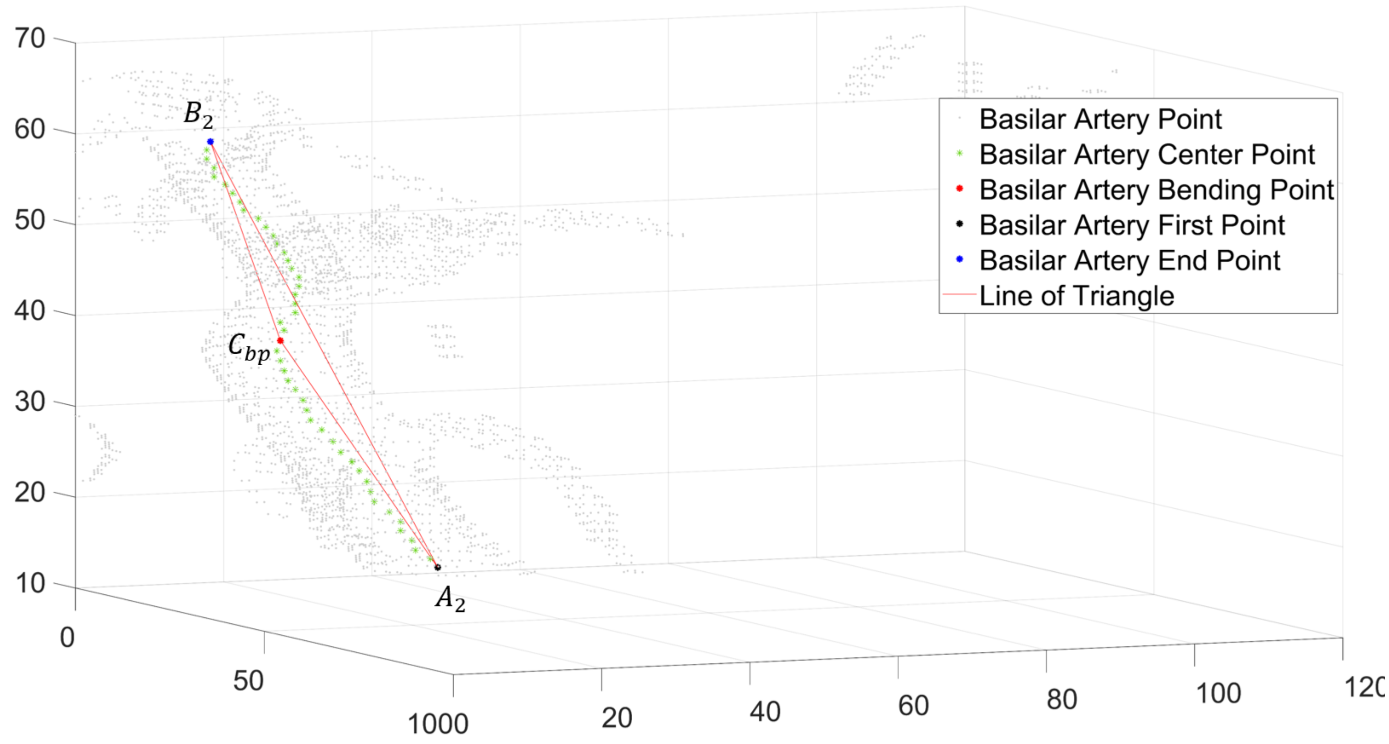

70
60
50
40
30
20
10
0
50
1000
20
40
60
80
100
B_2
C_{bp}
A_2
Basilar Artery Point
Basilar Artery Center Point
Basilar Artery Bending Point
Basilar Artery First Point
Basilar Artery End Point
Line of Triangle

(av)

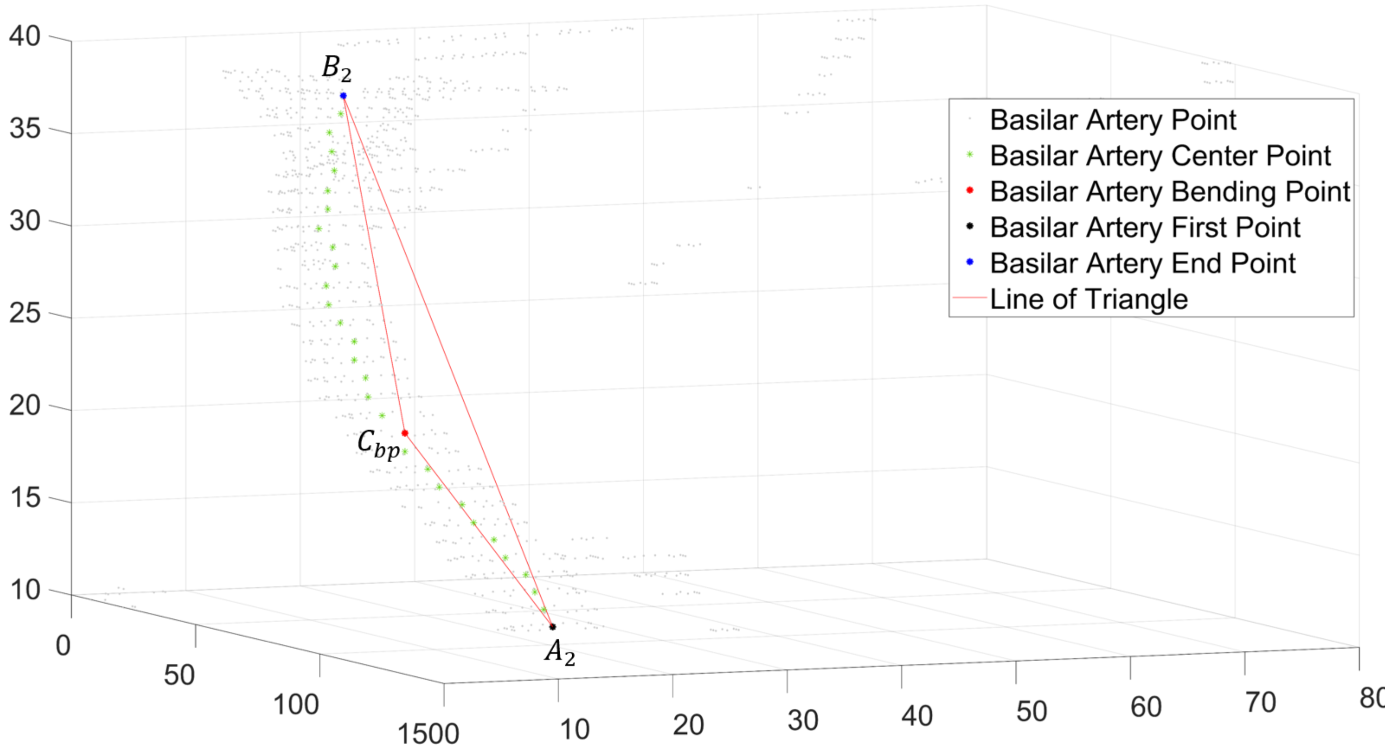

(aw)

Figure S1: Angle measurement plot of basilar artery. (a) Person E, (b) Person F, (c) Person G, (d) Person H, (e) Person I, (f) Person J, (g) Person K, (h) Person K, (i) Person M, (j) Person N, (k) Person O, (l) Person P, (m) Person Q, (n) Person R, (o) Person S, (p) Person T, (q) Person U, (r) Person V, (s) Person W, (t) Person X, (u) Person Y, (v) Person Z, (w) Person AA, (x) Person AB, (y) Person AC, (z) Person AD, (aa) Person AE, (ab) Person AF, (ac) Person AG, (ad) Person AH, (ae) Person AI, (af) Person AJ, (ag) Person AK, (ah) Person AL, (ai) Person AM, (aj) Person AN, (ak) Person AO, (al) Person AP, (am) Person AQ, (an) Person AR, (ao) Person AS, (ap) Person AT, (aq) Person AU, (ar) Person AV, (as) Person AW, (at) Person AX, (au) Person AY, (av) Person AZ, (aw) Person BA,

We define people E, K, L, N, P, S, V, X, AE, AJ, AL, AN, AO, AP, AQ, AU as group A. It can be confirmed that the bending of the basilar artery is relatively normal as the angle of C_{bp} is wide. We define people F, Q, R, T, U, AM, AT, AV, AZ, BA as group B. The angle of C_{bp} in group B is less than that in group A, indicating that the bending of the basilar artery in group B is progressing more than in group A. Similarly, when people G, H, I, J, M, O, W, Y, Z, AA, AB, AC, AD, AF, AG, AH, AI, AK, AR, AS, AW, AX, AY are defined as group C, the angle of C_{bp} in group C is less than those in the other groups. This means that the bending of the basilar artery in the group C is most severe. However, the reason why the angle of C_{bp} in the figures of group C seems to be large is because the axes in the figures of group C is rotated to match those in the figures in the other groups.
